# Reference data for body composition parameters in normal-weight Polish adolescents: results from the population-based ADOPOLNOR study

**DOI:** 10.1007/s00431-024-05736-8

**Published:** 2024-09-26

**Authors:** Maria Kaczmarek, Magdalena Durda-Masny, Tomasz Hanć

**Affiliations:** Faculty of Biology, Institute of Human Biology and Evolution, Adam Mickiewicz University, Uniwersytetu Poznańskiego 6, 61-614 Poznań, Poland

**Keywords:** Body composition parameters, Adolescents, Bioelectrical impedance analysis, Lambda mu-sigma (LMS) method, Centile values, Centile curves

## Abstract

The aim of this study was to develop age- and sex-specific reference data for body composition parameters, including fat-free mass (FFM), body cell mass (BCM), fat mass (FM) and related indices, fat mass percentage (FM%), total body water (TBW) and muscle mass (MM) and to assess differences in these parameters between male and female adolescents. Bioelectrical impedance analysis (BIA) data were obtained from an ethnically homogeneous sub-sample of 4037 normal-weight participants selected on the basis of BMI from the total sample (2005 males and 2032 females aged 10–18 years) of the ADOPOLNOR study, a cross-sectional population-based survey conducted in western Poland. The LMS method and RefCurv version 0.4.2 software package were used to calculate centile values and plot smoothed centile curves. The results showed age-related changes in body composition parameters and a clear sexual dimorphism. From the age of 13 (for FFM) or 14 (for BCM, BCMI and MM) or throughout adolescence (for TBW and FFMI), boys had consistently higher values for these parameters than girls (*p* < 0.01 for each). However, they had significantly lower values for FM, FMI and FM% (1.3, 1.6 and 1.5 times lower at 18 years, respectively), indicating a lower proportion of body fat than girls (*p* < 0.01 for each). The centile values and curves were specific for boys and girls of all ages. Compared with other European adolescent cohorts, the data fit the normal-weight subsamples better than the population reference data.

*Conclusion*: This study provides reference data for body composition in normal (healthy) weight Polish adolescents, providing a contextualised framework that takes into account the unique physiological and environmental factors of the region. These reference data may also be useful for other Central European adolescents.

**Table:** 

**What is Known:** *• Adolescence is a period of significant physical growth and increased susceptibility to nutritional problems due to factors such as suboptimal nutrition, unhealthy dietary choices, inadequate physical activity and psychosocial stressors related to body image concerns.* *• Body composition (BC) parameters, rather than BMI alone, are more effective in identifying nutritional abnormalities and health risks such as eating disorders, obesity and chronic diseases. Existing studies provide reference data for BC parameters, but often do not cover all relevant measures or specific regional populations.*	
**What is New:** *• This study provides new age- and sex-specific reference values for a comprehensive range of body composition parameters, including body cell mass (BCM), total body water (TBW) and muscle mass (MM), in addition to fat-free mass (FFM) and fat mass (FM), in a large, ethnically homogeneous, normal-weight population of Polish adolescents aged 10–18 years.* *• These reference values for the normalweight cohort reflect healthy adolescent growth and body composition patterns, minimising bias from abnormal weight status. In addition, they can be applied to other Central European adolescent populations.*

**Supplementary Information:**

The online version contains supplementary material available at 10.1007/s00431-024-05736-8.

## Introduction

Adolescence (10–19 years) is typically a period of robust vitality, marked by peak physical maturity, increased strength, speed, reaction time and improved immune function [1]. Paradoxically, however, this period also coincides with increased susceptibility to nutritional problems. Inadequate nutrition, unhealthy dietary choices, inadequate physical activity and various psychosocial factors such as emotional stressors, body image concerns and societal pressures to conform to beauty ideals exacerbate this vulnerability [2, 3]. Effective strategies, including awareness and surveillance, are essential to address these nutritional disorders [4]. Although BMI is commonly used metric, it has limitations in assessing body fat distribution and hidden deficiencies [5]. As a result, body composition (BC) parameters are more effective in identifying abnormalities such as eating disorders [6], obesity [7], delayed or stunted growth [8], and chronic diseases such as cystic fibrosis, renal disease or inflammatory bowel disease, as well as muscle wasting [9, 10, 11]. Regular assessment using updated reference data is essential for early intervention in the nutritional status of adolescents. Several reference sets have been developed for BC parameters, expressed as age-, sex- and ethnic-specific standard deviations, centile values and growth charts. Recent developments include paediatric reference curves and the corresponding centile values for children aged 8 to 20 years [12]. Data from whole-body dual-energy X-ray absorptiometry (DXA) scans in the National Health and Nutrition Examination Survey (NHANES) provide key measures, including total bone mass density (BMD) and bone mineral content (BMC) [12], as well as fat mass index (FMI) and lean body mass index (LBMI) [13]. In addition, reference centiles for body fat percentage (FM%), FMI and LBMI are now available for different DXA systems such as e Prodigy and iDXA GE Healthcare Lunar systems [14]. Recently published reference values for FM%, FMI and trunk/limb FM assessed by dual-energy X-ray absorptiometry (DXA) are available for a large cohort of Central European children and adolescents aged 6–18 years from the LEAD project [15]. Schmidt and colleagues used bioelectrical impedance analysis (BIA) to ascertain reference values for fat-free mass index (FFMI), fat mass index (FMI), body cell mass index (BCMI), and phase angle (PA) in a nationwide cohort of participants aged 4–24 years in Germany as part of the Motoric Module Study Wave 1 [16]. The study highlighted significant differences in body composition between the representative German sample and the normal-weight sub-sample, emphasising the need for personalised reference values to address specific challenges in adolescent body composition.

Despite the abundance of reference data for body measurements, including body height and weight, BMI, waist and hip circumference and skinfold thickness [17, 18, 19], there is a significant dearth of reliable reference data for complete BC parameters in Polish adolescents. Although Austrian data provide valuable information on lean and fat mass, they do not cover all relevant BC parameters. Therefore, the objective of this study is twofold: firstly, to establish reference data for body composition parameters in Polish adolescents representative of Central Europe, and secondly, to investigate sex differences in these parameters.

## Materials and methods

### Study design and participants

The data used in this analysis were obtained from the ADOPOLNOR study, a cross-sectional survey designed to investigate the adolescents’ health and quality of life. The study comprised a random sampling methodology to recruit a representative cohort of students aged 10 to 18 years from primary and secondary schools in the Wielkopolska province, situated in the west of Poland. All participating students were of Polish origin and from families representing all social strata and residential areas in Poland. The study design and protocol were approved by the Bioethics Commission of the Medical University of Poznań (Resolution No. 311/07) and the Regional Board of Education of Poznań (Resolution No. WAF-405/1/JM/07). In accordance with the ethical standards for research practice, written informed consent was obtained from all participants, including parental or guardian consent for students under the age of 16, joint consent from both the student and their parent or guardian for those aged 16 but not yet 18 and individual consent from students aged 18 and older.

The study protocol included medical examinations, anthropometric measurements and the administration of questionnaires to collect background information from parents and self-reports. All examinations were carried out in school nurseries during the morning hours by well-trained staff in compliance with the Declaration of Helsinki and its subsequent amendments.

### Measures

The standing body height and weight of the participants were measured in accordance with standardised procedures using a Swiss Gneupel Precision Mechanics (GPM) portable anthropometer (accuracy of 0.1 cm) and a calibrated electronic precision health scale (Radwag 100/200 OW, Radom, Poland; accuracy: 0.1 kg) [20]. Body mass index (BMI) was calculated as weight (kg) divided by height squared (m^2^) and was used to categorise weight status according to Cole’s age- and sex-specific BMI cut-off points for children and adolescents recommended by the International Obesity Task Force (IOTF) [21, 22].

Bioelectrical impedance analysis (BIA) was used to assess body components. Participants were tested in the morning hours. They had to fast for at least 2 h and emptied their bladders before testing. Measurements were taken with participants lying flat on their back in a tetrapolar configuration using a BIA 101 device (AKERN, Florence, Italy) and following the manufacturer’s guidelines (https://www.akern.com/en), with data analysed using BODYGRAM software (version 1.31). The following whole-body parameters were measured: fat-free mass (FFM), body cell mass (BCM), muscle mass (MM) and fat mass (FM) in kilograms (kg), as well as FM as a percentage of total body weight (FM%) and total body water (TBW) in litres (L). Additionally, the following indices were calculated: FFMI (FFM/height^2^), FMI (FM/height^2^) and BCMI (BCM/height^2^).

### Data analysis

Of the 5482 participants in the ADOPOLNOR study, only those whose weight status was within the normal (healthy) range, as defined by Cole’s age- and sex-specific BMI cut-off points for children and adolescents, were selected for further analysis. This subset consisted of 4037 participants, including 2005 male adolescents (68.8% of the male sample) and 2032 female adolescents (71.9% of the female sample) aged 10 to 18 years.

The data were processed using RefCurv version 0.4.2 software (https://refcurv.com) which operates in the R programming language with the Generalised Additive Models for Location Scale and Shape (GAMLSS) package [23]. The RefCurv software generates centile values and plots tailored to paediatric reference curves using the Lambda-Mu-Sigma (LMS) method to address issues of unequal variance and skewness in growth data [24]. The software selects the degrees of freedom for the L, M and S splines based on the Bayesian information criterion (BIC), thereby ensuring the generation of smooth centile curves. The centile values were calculated based on the selected effective degrees of freedom (edf) and subsequently used to construct plots representing the distribution of body composition metrics by age and sex.

Descriptive statistics were calculated using STATISTICA 13.3, with sex differences determined using appropriate statistical tests and assuming a critical value for a significance level of *α* < 0.05.

Additional information on measures and methods can be found in the supplementary material (File S1. Mat&Met).

## Results

Table 1 presents descriptive data on height, weight, BMI and body composition (BC) parameters for normal-weight male and female adolescents aged 10–18 years.

**Table 1:** Descriptive statistics of body composition parameters in normal-weight Polish adolescents stratified according to sex and age

Variable	Age (years)								
	10	11	12	13	14	15	16	17	18
Boys									
Sample size, *n*	216	207	212	226	226	209	237	222	250
Height (cm)	143.6 ± 6.6	145.8 ± 5.8	152.4 ± 6.7	160.0 ± 7.9	168.4 ± 8.7^a^	171.4 ± 7.3^a^	175.8 ± 7.4^a^	178.8 ± 6.8^a^	179.3 ± 6.1^a^
Weight (kg)	35.4 ± 4.7	36.9 ± 4.9	41.9 ± 5.6	47.7 ± 6.8	54.5 ± 7.7^a^	57.8 ± 7.3^a^	63.2 ± 7.6^a^	67.2 ± 7.3^a^	69.6 ± 6.7^a^
BMI (kg/m^2^)	17.1 ± 1.3	17.3 ± 1.5	18.1 ± 1.6	18.5 ± 1.6	19.2 ± 1.6^a^	19.7 ± 1.7	20.4 ± 1.6^a^	21.0 ± 1.6^a^	21.7 ± 1.7^a^
FFM (kg)	28.3^a^ 25.4;30.9	29.7 27.1;32.7	33.7 30.1;37.0	39.4 34.5;43.6	44.8^a^ 40.6;50.0	47.8^a^ 43.5;53.0	53.0^a^ 49.5;57.3	56.6^a^ 52.2;60.9	57.5^a^ 53.8;61.7
BCM (kg)	13.7 11.9;15.7	14.7 12.8;16.4	16.0 14.5;18.1	18.9 16.6;21.9	22.2^a^ 19.9;25.6	24.7^a^ 22.1;27.7	27.5^a^ 25.5;30.6	29.9^a^ 27.3;32.4	30.6^a^ 28.4;33.7
TBW (L)	24.8^a^ 21.9;27.0	26.1^a^ 24.4;28.0	28.6^a^ 26.3;30.5	32.2^a^ 29.0;34.8	35.6^a^ 33.0;38.8	37.5^a^ 32.6;40.9	39.8^a^ 36.8;42.4	41.2^a^ 37.9;44.3	41.7^a^ 39.2;44.9
MM (kg)	17.0 14.9;19.5	18.3 16.1;20.3	19.5 18.0;22.5	23.8 20.5;27.2	27.9^a^ 24.9;31.7	30.4^a^ 27.5;34.2	34.92^a^ 31.8;37.7	36.9^a^ 33.7;39.5	37.7^a^ 35.2;41.1
FM (kg)	6.8 4.3;8.9	7.0^a^ 4.5;8.4	7.7 5.8;10.7	8.0^a^ 5.5;10.5	8.2^a^ 6.1;11.2	8.6^a^ 6.3;11.7	9.6^a^ 7.3;12.3	10.0^a^ 7.4;13.0	11.8^a^ 9.3;15.3
FM (%)	19.4^a^ 12.9;23.2	17.9^a^ 13.1;22.6	17.0 12.5;22.6	16.9^a^ 11.9;22.0	15.5^a^ 11.4;20.5	15.8^a^ 11.2;20.0	16.0^a^ 12.4;19.3	15.5^a^ 11.5;19.2	16.7^a^ 13.4;20.0
FFMI (kg/m^2^)	13.9^a^ 12.8;14.6	14.0 13.2;14.9	14.6 13.6;15.2	15.1^a^ 14.4;16.2	16.1^a^ 15.0;17.1	16.4^a^ 15.3;17.6	17.2^a^ 16.1;18.0	17.5^a^ 16.6;18.6	17.8^a^ 16.7;18.9
BCMI (kg/m^2^)	6.7 5.9;7.5	6.8 6.2;7.6	6.9 6.3;7.7	7.4 6.7;8.3	7.9^a^ 7.3;8.8	8.4^a^ 7.6;9.3	9.0^a^ 8.2;9.8	9.3^a^ 8.6;1011	9.5^a^ 8.7;10.4
FMI (kg/m^2^)	3.2 2.2;4.1	3.2^a^ 2.0;3.8	3.4 2.6;4.4	3.2^a^ 2.1;4.2	2.9^a^ 2.1;4.0	3.0^a^ 2.2;4.1	3.2^a^ 2.3;4.1	3.1^a^ 2.4;4.1	3.7^a^ 2.9;4.6
Girls									
Sample size, n	216	228	203	208	217	250	258	209	243
Height (cm)	142.2 ± 6.6	145.1 ± 6.6	151.8 ± 8.2	159.9 ± 6.8	162.7 ± 5.6	163.8 ± 5.9	165.3 ± 6.4	165.4 ± 5.8	165.5 ± 6.3
Weight (kg)	34.7 ± 4.8	36.3 ± 5.6	42.2 ± 7.0	48.8 ± 6.0	52.3 ± 5.9	53.2 ± 5.4	56.9 ± 6.2	57.3 ± 6.0	58.2 ± 6.4
BMI (kg/m^2^)	16.9 ± 1.4	17.3 ± 1.5	18.2 ± 1.6	19.0 ± 1.5	19.8 ± 1.7	19.9 ± 1.5	20.8 ± 1.6	20.9 ± 1.6	21.3 ± 1.7
FFM (kg)	26.7 24.6;29.7	29.7 27.0;32.9	32.9 29.6;37.2	37.5 34.7;41.1	40.2 36.7;43.2	40.5 37.4;42.8	42.0 39.2;45.2	42.0 39.6;44.2	42.0 39.5;44.9
BCM (kg)	12.7 11.7;14.8	14.5 12.7;16.5	16.3 13.5;18.7	18.3 16.3;20.9	20.1 17.7;22.7	20.5 18.5;22.8	21.6 19.5;23.7	21.2 19.6;23.3	21.3 19.7;23.7
TBW (L)	22.4 20.7;24.0	24.4 22.6;26.1	26.0 24.0;28.4	28.4 26.8;30.5	29.8 27.8;31.7	29.9 28.1;31.4	30.7 28.7;32.9	30.6 28.9;32.4	30.7 28.6;32.8
MM (kg)	15.7 14.5;18.3	18.0 15.8;20.6	20.3 17.0;23.5	22.8 20.2;26.0	25.0 22.1;27.9	25.2 23.0;27.9	26.8 24.2;29.4	26.3 24.4;28.7	26.5 24.6;29.9
FM (kg)	7.0 5.5;8.9	7.3 5.6;8.9	9.0 6.3;11.2	10.2 8.0;13.3	12.1 9.7;14.9	13.1 10.7;15.5	14.7 12.4;17.1	14.6 12.3;17.7	15.3 12.7;19.0
FM (%)	20.8 17.6;23.8	19.9 16.0;23.3	21.4 16.3;25.9	21.9 17.7;26.1	23.6 19.6;27.5	25.1 20.6;28.3	25.6 22.8;28.6	26.1 22.1;29.5	27.0 23.1;31.0
FFMI (kg/m^2^)	13.2 12.4;14.1	13.7 12.8;14.5	14.3 13.3;15.2	14.7 14.0;15.5	15.0 14.2;16.1	15.1 14.2;15.8	15.3 14.6;16.3	15.4 14.7;16.2	15.4 14.8;16.1
BCMI (kg/m^2^)	6.2 5.8;6.9	6.6 6.0;7.6	6.9 6.2;7.8	7.2 6.6;7.9	7.5 6.9;8.6	7.7 6.9;8.4	7.8 7.1;8.5	7.8 7.3;8.4	7.8 7.3;8.6
FMI (kg/m^2^)	3.4 2.9;4.2	3.5 2.7;3.9	3.9 2.8;4.8	4.1 3.2;5.2	4.6 3.8;5.5	4.9 3.9;5.8	5.4 4.5;6.2	5.4 4.4;6.4	5.6 4.7;6.7

The table shows mean ± standard deviation; median and Q_1_; Q_3_. Abbreviations used: *BMI* body mass index, *FFM* fat-free mass, *BCM* body cell mass, *TBW* total body water, *MM* muscle mass, *FM* fat mass, *FFMI* fat-free mass index, *FMI* fat mass index, *BCMI* body cell mass index; ^a^significant difference between boys and girls of the same age at *p* < 0.05

As expected, boys were generally taller and heavier than girls, with significant differences appearing at age 14 (*p* < 0.01) and continuing into late adolescence. At age 18, boys were on average 13.8 cm taller and 11.7 kg heavier than girls. The BMI of both boys and girls increased with age, with significant differences emerging at age 13 (*p* < 0.05), although the difference remained small. At the age of 18, boys had a slightly higher average BMI than girls (21.7 vs. 21.3 kg/m^2^). Boys had consistently higher values for FFM, BCM, TBW and MM than girls, with significant differences (*p* < 0.01) starting at age 13 (for FFM), 14 (for BCM, BCMI and MM) or throughout adolescence (for TBW and FFMI). In contrast, girls had higher values of FM, FMI and FM% indicating a higher proportion of body fat (*p* < 0.01). Notably, FM% decreased with age in boys but increased in girls although, absolute FM increased in both sexes. At the age of 18 years, boys and girls showed significant differences in FFM (57.5 vs. 42.0 kg), BCM (30.6 vs. 21.3 kg), TBW (41.7 vs. 30.7 L) and MM (37.7 vs. 26.5 kg). Specifically, girls had about 1.3 times more FM than boys (15.3 kg vs. 11.8 kg) and about 1.5 times more FM% than boys (27.0% vs. 16.7%).

Throughout adolescence, there was a consistent difference in FMI between boys and girls (*p* < 0.01). The difference, which was small between 10 and 11 years of age, gradually increased with age. Boys’ FMI remained stable between the ages of 10 and 14 (3.2 kg/m^2^ at age 10 to 2.9 kg/m^2^ at age 14), whereas girls’ FMI increased more significantly, reaching 4.6 kg/m^2^ at age 14. At the age of 18, girls had a 1.6 times higher FMI than boys (5.6 kg/m^2^ vs. 3.7 kg/m^2^). The increase in FFMI was consistently higher in boys, from 13.9 kg/m^2^ at age 10 to 17.8 kg/m^2^ at age 18, while girls’ FFMI increased from 13.2 kg/m^2^ at age 10 to 15.4 kg/m^2^ at age 18. Body cell mass index (BCMI) also increased in both sexes, with significant differences appearing at age 15 and peaking at 1.7 kg/m^2^ at age 18.

**Fig. 1:**
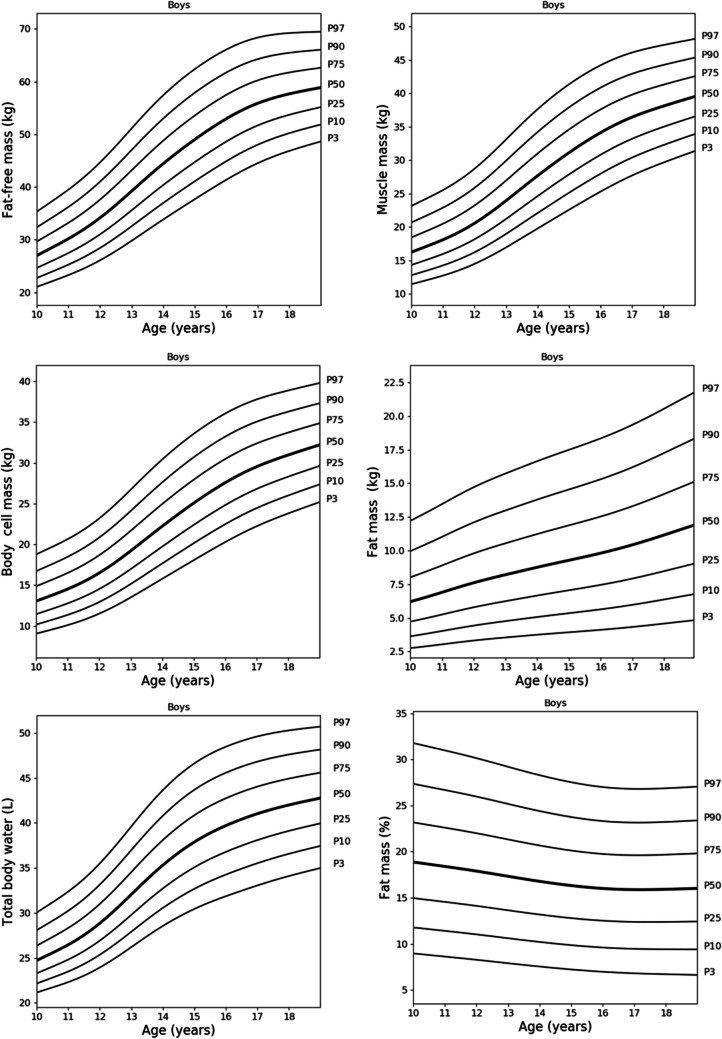
Reference centile curves for fat-free mass, body cell mass, total body water, muscle mass, fat mass and fat mass percentage in Polish boys aged 10 to 18 years smoothed by the LMS method. The numbers on the right-hand side represent the 3rd, 10th, 25th, 50th, 75th, 90th and 97th centiles

**Fig. 2:**
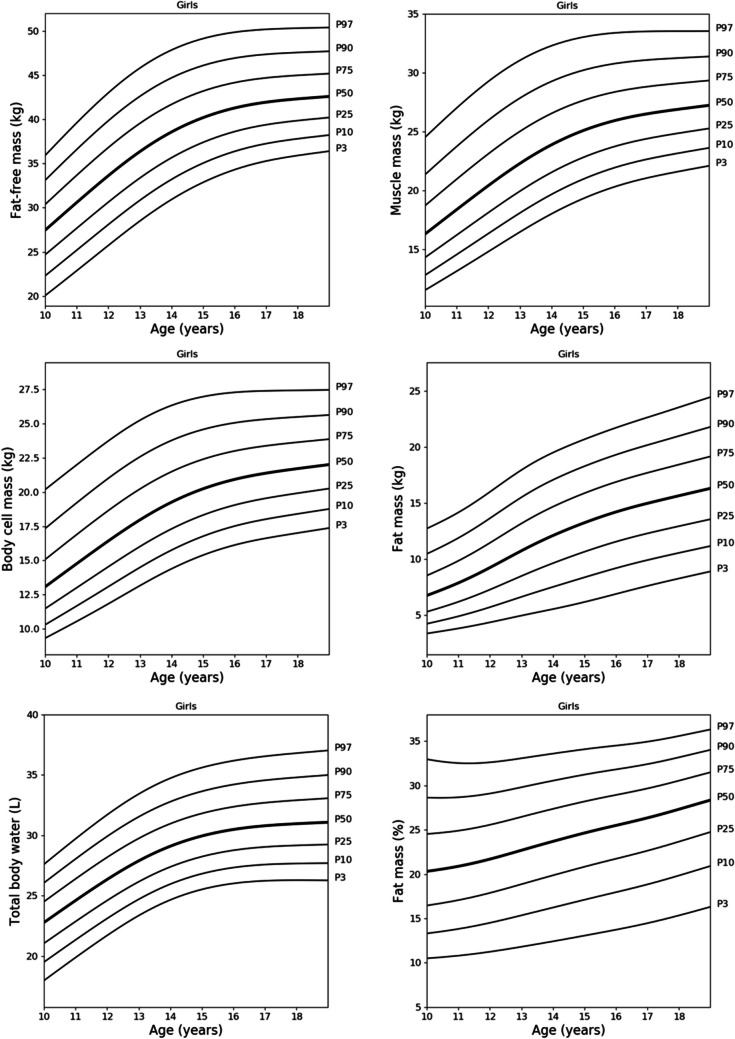
Reference centile curves for fat-free mass, body cell mass, total body water, muscle mass, fat mass and percentage of fat mass in Polish girls aged 10 to 18 years smoothed by the LMS method. The numbers on the right-hand side represent the 3rd, 10th, 25th, 50th, 75th, 90th and 97th centiles

**Fig. 3:**
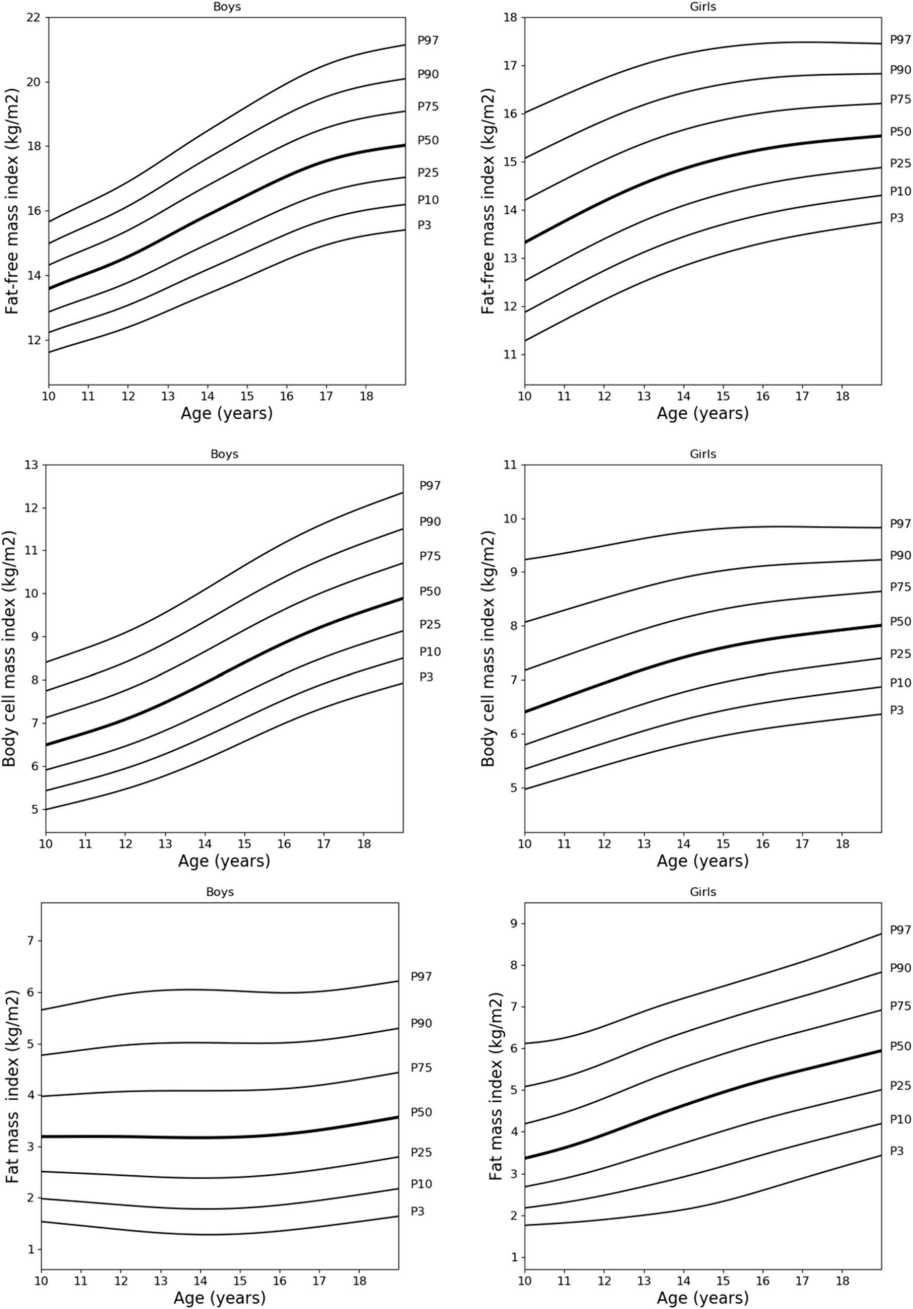
Reference centile curves for fat-free mass index, body cell mass index and fat mass index in Polish boys (left) and girls (right) aged 10 to 18 smoothed by the LMS method. The numbers on the right-hand side represent the 3rd, 10th, 25th, 50th, 75th, 90th and 97th centiles

The supplementary files include centile values for BC parameters for boys and girls aged 10 to 18 years, detailed in Tables S1 and S2. Figures 1, 2, and 3 show smoothed centile curves for boys (Fig. 1), girls (Fig. 2) and FFM, BCM and FM indices for both sexes (Fig. 3).

## Discussion

This study presents new age- and sex-specific reference values and charts for various body composition parameters in a large, ethnically homogeneous, normal-weight population of Polish adolescents aged 10–18 years. Moreover, these reference values may serve as benchmarks for other Central European populations. In order to establish healthy ranges and ensure accurate reference points, individuals who were underweight, overweight or obese were excluded from the analysis, as their inclusion could potentially result in skewing the results due to lower lean mass or excess adiposity. Both abnormal weight statuses may bias reference data towards lower/higher extremes and fail to represent the typical healthy adolescent population. Significant discrepancies in FFM and FM were identified between normal-weight and underweight, overweight or obese study participants (data currently undergoing analysis), thereby substantiating the utilisation of a homogeneous, normal-weight cohort to establish baseline references for typical adolescent growth and body composition. This study uniquely includes measures such as BCM, TBW and MM in addition to the more commonly used FFM and FM, thereby improving our understanding of adolescent body composition for screening and clinical purposes.

The results show that body composition measures generally increase with age, reflecting overall growth during adolescence, with boys experiencing a decrease in FM% due to faster lean mass growth during puberty [25]. Boys typically have higher cell mass, muscle mass and water content, while girls have higher FM, FMI and FM%, consistent with differences attributed to genetics, hormones and lifestyle factors during adolescence [26]. During this period, boys experience a surge in testosterone production, which promotes muscle growth and reduces body fat, while girls experience increased levels of oestrogen, which leads to fat accumulation, particularly around the hips and thighs [27].

Although there are generally accepted patterns of growth during adolescence, the timing and intensity of growth spurts can vary significantly between individuals. Some may experience growth spurts earlier or later, with variations in the rate of growth and the timing of sexual maturation stages. Consequently, there is considerable individual variation in body composition parameters, both within and between sexes. It is therefore essential to consider these variations when interpreting findings related to growth during adolescence, as they emphasise the uniqueness of each individual's development during this transitional period.

A comparative analysis of the reference centile values for FFMI and FMI in Polish normal-weight adolescents with data from Austria (LEAD study) [15], the United Kingdom [28], Germany (MoMo study population data) [16] and Tyrol (EVA4YOU study) [29] shows similar overall patterns, with notable differences such as significantly lower 97th (98th in UK) centile values in our dataset, probably due to exclusion of overweight and obese individuals. Raw centile curves for the 3rd (2nd in UK cohort), 50th and 97th (98th in UK cohort) are available in the supplementary material in Figure S1. Raw centile curves for the 3rd, 50th and 97th of our data and the normal-weight subsample of German adolescents (Figure S2 in the supplementary material) demonstrate similar growth trajectories, but with more marked differences for boys. These findings underscore the value of using normal-weight adolescents as a reference benchmark for body composition parameters.

The study addresses the potential impact of diverse measurement techniques on the accuracy of body composition data. The LEAD and UK cohort studies employed DXA, whereas the MoMo and EVA4YOU studies utilised BIA, the same techniques as employed in the present study. BIA is simple, economical and non-invasive method, making it well-suited for population surveys and outpatient clinical applications. Achamrah and colleagues identified only minor discrepancies between these methods when studying over 3000 adult patients, particularly in those with a BMI between 16 and 18 kg/m^2^ [30]. This suggests that the choice of measurement method does not significantly affect comparative results at a population level. However, they found significant discrepancies when assessing individuals, independent of BMI.

The establishment of new reference data for Polish adolescents gives rise to the question of whether local reference data should be prioritised over international benchmarks. The existing literature provides consistent support for the use of local growth references for the monitoring of changes in physical growth during childhood and adolescence [31, 32, 33]. The study conducted by Kułaga and colleagues demonstrates the superiority of local references in capturing population-specific variation in physical growth in Polish children and adolescents compared to international benchmarks [34]. We think that local references are able to account for population-specific variation and provide accurate benchmarks that are tailored to the specific characteristics of the local population. This is particularly important for understanding and monitoring growth patterns and health metrics in specific populations, ensuring that assessments and interventions are appropriately tailored to the distinctive genetic, environmental and cultural factors that influence a local group. Conversely, international references facilitate cross-population analysis and consistency in research, which is essential for clinical practice and global health initiatives. They allow comparisons to be made between different regions and populations, thereby highlighting broader trends and inequalities in health and development. This consistency is essential for multinational studies, global health assessments and the formulation of universal health guidelines. Both types of data are valuable, and the choice depends on the purpose of the study. Local references are ideal for accurate population-specific assessments and interventions, while international references are essential for comparative studies and the establishment of universal health standards.

A key element of body composition assessment is the establishment of cut-off points to facilitate the identification of individuals at risk of various health outcomes. These points serve as critical markers for distinguishing between normal and abnormal body composition. There is no consensus on the optimal cut-off points for these measurements, and recommendations vary between studies. McCarthy and colleagues propose the use of fat mass cut-off points in the second, 85th and 95th centiles to define the underfat, normal, overfat and obese categories, respectively [35]. While there is consensus on the definitions of overweight and obese, it is recommended that the underfat category be defined using the 5th centile rather than the 2nd [36]. It is preferable to establish cut-off points based on the risk of various diseases rather than arbitrary choices based solely on centile thresholds [29, 37]. Given the disparate implications of body composition parameters for health, future research will seek to establish centile values for each parameter, thereby facilitating a more nuanced understanding of healthy and pathological body composition.

### Study limitations

The present study offers valuable insights into body composition during adolescence; however, it is not without limitations. The cross-sectional design does not allow for tracking of individual changes over time. The lack of data on sexual maturation stages affects the ability to fully interpret changes in muscle mass and fat accumulation during puberty. Sexual maturation is significantly correlated with body composition, with adolescents of the same age group but at different stages of sexual maturation showing differences in body components. Nevertheless, chronological age remains a useful indicator due to its close relationship with the stages of sexual maturation. Tanner’s work showed that despite individual variability, the general pattern of maturation correlates with chronological age [38]. Consequently, age-based references can still provide valuable insights into typical growth and development. Many studies in this field, including those referred to in this paper, also use chronological age for pragmatic and ethical reasons.

Despite the limitations outlined above, the main strengths of this study include the use of a cross-sectional study design, which is well suited to population-based surveys and ensures that the results are generalisable to the wider population [39]; the use of a large, randomly selected population-based sample; a sufficiently large number of participants across all adolescent age groups, which increases the reliability of our findings; comprehensive body composition measurements and adherence to relevant standards [40, 41]. Although our sample does not represent the entire cohort of Polish adolescents, it includes all social strata and residential areas typical of Poland, thus ensuring broad representativeness. Further refinement of these references may be achieved by future studies including stages of sexual maturation. However, the age-based approach used in this study is a valuable contribution in itself.

## Conclusion

The establishment of reference data for body composition in Polish adolescents is of significant scientific importance. These local references account for unique regional physiological and environmental factors, thereby providing a valuable framework for research, screening and clinical assessment. Such data facilitate the early detection and intervention of nutritional deficiencies, promote healthier adolescent development and reduce long-term health risks. Furthermore, these references may serve as a useful reference data for other Central European adolescents.

## Supplementary Information

Below is the link to the electronic supplementary material.

Supplementary file1 (DOCX 19.1 KB)

Supplementary file2 (DOCX 91.2 KB)

Supplementary file3 (DOCX 92.2 KB)

Supplementary figure 1. Comparison of the raw 3rd (2nd in Britain cohort), 50th, and 97th (98th in Britain cohort) reference centile curves for FFMI and FMI in normal-weight Polish adolescents with population data from Austrian (LEAD) [15], United Kingdom [28], German (MoMoP) [16] and Tyrolean (EVA4YOU) adolescents [29]. (PNG 1740 KB)

High resolution image (TIF 306 kb)

Supplementary figure 2. Comparison of the raw 3rd, 50th, and 97th reference centile curves for FFMI and FMI in normal-weight Polish and normal-weight German adolescents (MoMoNW) [16]. (PNG 1130 KB)

High resolution image (TIF 211 kb)

## Data Availability

No datasets were generated or analysed during the current study.

## Abbreviations

BC: Body composition
BIA: Bioelectrical impedance analysis
BCM: Body cell mass
BCMI: Body cell mass index
DXA: Dual energy X-ray absorptiometry
FM: Fat mass
FM%: Percentage of fat mass
FMI: Fat mass index
FFM: Fat-free mass
FFMI: Fat-free mass index
MM: Muscle mass
LMS method: Lambda-mu-sigma method
TBW: Total body water
