# Supplementary material for: Reference data for body composition parameters in normal-weight Polish adolescents: results from the population-based ADOPOLNOR study
Source: Eur J Pediatr. 2024 Sep 26;183(11):5021–31. doi: 10.1007/s00431-024-05736-8 (PMC11473596; doi:10.1007/s00431-024-05736-8)
Supplement: Supplementary file 1 — Supplementary file1 (DOCX 19.1 KB) [file 431_2024_5736_MOESM1_ESM.docx]

# Materials and Methods

*Measures* Anthropometric measurements were taken according to a standard procedure [20]. Participants, wearing light gym clothing and no shoes, stood in an upright position with their heels together, arms at the sides, legs straight, shoulders relaxed and head in the Frankfurt plane. Standing height was measured to the nearest 1 mm using a portable Swiss-made GPM anthropometer, from the highest point of the midline vault (*vertex*) to the floor on which the participant stood (*basis*). Body weight was measured to the nearest 0.1 kg using a calibrated electronic scale. Body mass index (BMI) was calculated as weight (kg) divided by height squared (m^2^) and was used to categorise weight status according to Cole's age- and sex-specific BMI cut-off points for children and adolescents recommended by the International Obesity Task Force (IOTF) [21, 22]. In our study, ‘normal weight’ was defined using these cut-off points, which are internationally accepted and are used to classify underweight, normal weight, overweight and obesity in children and adolescents aged 2-18 years. Specifically, Cole's cut-off points provide a standardised method for determining BMI percentiles that correspond to the adult BMI categories of underweight (BMI < 18.5), normal weight (BMI 18.5-24.9), overweight (BMI 25-29.9) and obesity (BMI ≥ 30) at different ages of growth. For our study, only participants whose BMI fell within the 'normal weight' range according to these cut-off points were included in further analysis. This approach ensures that the categorisation of weight status is age-appropriate and comparable across different populations.

Bioelectrical impedance analysis (BIA) was used to assess a number of body components. Prior to testing, participants were instructed not to eat or drink for at least two hours and to empty their bladders. Testing was performed in the morning hours. Whole-body impedance was measured using the BIA 101 new edition (AKERN, Florence, Italy), a phase-sensitive single-frequency analyser (SF-BIA) based on the 4-compartment model, according to the manufacturer's guidelines (https://www.akern.com/en).

The instrument is operated with an alternating AC current of 400 µA at a frequency of 50 kHz. To ensure accuracy and consistency, the instrument was calibrated at least twice a year using the standard control circuit provided by the manufacturer. The control circuit had a known impedance (Rz) of 380 ohms and a reactance (Xc) of 47 ohms. The instrument had an accuracy of ±1% for Rz and ±2% for Xc. The data were analysed using dedicated software, specifically BODYGRAM version 1.31 (AKERN, Florence, Italy, 2016).

Measurements were taken in a tetrapolar setup after the participants had been lying flat with their arms and legs relaxed for at least 10 minutes. The skin was cleaned with isopropyl alcohol before two low-impedance Ag/AgCl electrodes (Biatrodes, AKERN Srl, Florence, Italy) were placed on the dorsal side of the hands and feet, 5 cm apart, to ensure balanced measurements and minimise potential errors.

*Missing data* Of the original 5,703 participants, 221 were excluded due to incomplete data, leaving a final sample size of 5,482. We assessed the pattern of missing data to determine whether it was missing completely at random (MCAR) or missing at random (MAR). Using SPSS Statistics 29, we first performed logistic regression analyses to explore the relationship between missingness and observed variables such as age, sex and BMI. The results suggested that missingness was related to these observed variables, indicating that the data were likely to be MAR. To confirm this, we performed Little's MCAR test, which resulted in a non-significant *p*-value, supporting the conclusion that the data were not completely random. To account for the MAR pattern, we used multiple imputation using the Missing Values Analysis module. This process generated five imputed datasets where missing values were replaced with estimated values based on the observed data patterns. Sensitivity analyses were performed by comparing the results from the imputed datasets with those from a full case analysis. The consistency of the results in these analyses, with *p*-values > 0.05, confirmed the robustness of our results.

*Data Analysis* Chronological age was calculated in decimal values by subtracting the date of birth from the date of examination. Age groups were defined by whole years; for example, the 10-year group included participants aged 10.00 to 10.99 years.

Normality of the characteristic distribution was assessed using the Shapiro-Wilk test implemented in STATISTICA version 13.3. The following *p*-values were obtained Height: boys *p*=0.0857, girls *p*=0.0721. Weight: boys *p*=0.0531, girls *p*=0.0564. BMI: boys *p*=0.0511, girls *p*=0.0527. For other characteristics *p*<0.05. Sex differences were determined using appropriate statistical tests, Student's t-test, Kruskal-Wallis or Mann's U-test, and assuming a critical value for a significance level of α < 0.05.

Note: References are numbered as in the manuscript
