# Supplementary material for: Reference data for body composition parameters in normal-weight Polish adolescents: results from the population-based ADOPOLNOR study
Source: Eur J Pediatr. 2024 Sep 26;183(11):5021–31. doi: 10.1007/s00431-024-05736-8 (PMC11473596; doi:10.1007/s00431-024-05736-8)
Supplement: Supplementary file 2 — Supplementary file2 (DOCX 91.2 KB) [file 431_2024_5736_MOESM2_ESM.docx]

Table S1. Centile reference values for body composition parameters in normal weight Polish boys aged 10 to18

| Age | LMS parameters | | Centiles | | | | | | |
| --- | --- | --- | --- | --- | --- | --- | --- | --- | --- |
| (years) | L | S | 3rd | 10th | 25th | M 50th | 75th | 90th | 97th |
|  | Fat-free mass (kg) | | | | | | | | |
| 10.0 | -0.301 | 0.137 | 21.01 | 22.69 | 24.58 | 26.94 | 29.59 | 32.27 | 35.24 |
| 10.5 | -0.224 | 0.139 | 22.07 | 23.89 | 25.92 | 28.44 | 31.26 | 34.10 | 37.21 |
| 11.0 | -0.131 | 0.140 | 23.38 | 25.37 | 27.58 | 30.31 | 33.34 | 36.37 | 39.66 |
| 11.5 | -0.056 | 0.142 | 24.62 | 26.77 | 29.16 | 32.08 | 35.31 | 38.51 | 41.97 |
| 12.0 | 0.013 | 0.143 | 26.08 | 28.41 | 30.99 | 34.13 | 37.58 | 40.97 | 44.62 |
| 12.5 | 0.074 | 0.143 | 27.75 | 30.29 | 33.08 | 36.45 | 40.14 | 43.75 | 47.61 |
| 13.0 | 0.128 | 0.143 | 29.61 | 32.35 | 35.35 | 38.96 | 42.89 | 46.72 | 50.79 |
| 13.5 | 0.186 | 0.142 | 31.94 | 34.91 | 38.15 | 42.03 | 46.22 | 50.28 | 54.57 |
| 14.0 | 0.234 | 0.140 | 33.86 | 37.00 | 40.41 | 44.46 | 48.83 | 53.03 | 57.44 |
| 14.5 | 0.285 | 0.137 | 35.73 | 39.01 | 42.55 | 46.74 | 51.22 | 55.51 | 59.98 |
| 15.0 | 0.340 | 0.134 | 37.57 | 40.96 | 44.59 | 48.88 | 53.42 | 57.74 | 62.22 |
| 15.5 | 0.410 | 0.129 | 39.73 | 43.21 | 46.92 | 51.25 | 55.81 | 60.11 | 64.54 |
| 16.0 | 0.466 | 0.124 | 41.46 | 44.98 | 48.71 | 53.04 | 57.56 | 61.80 | 66.15 |
| 16.5 | 0.516 | 0.119 | 43.07 | 46.60 | 50.31 | 54.59 | 59.05 | 63.20 | 67.43 |
| 17.0 | 0.563 | 0.114 | 44.50 | 47.99 | 51.66 | 55.86 | 60.21 | 64.24 | 68.34 |
| 17.5 | 0.611 | 0.108 | 45.71 | 49.16 | 52.74 | 56.84 | 61.06 | 64.95 | 68.89 |
| 18.0 | 0.676 | 0.103 | 46.94 | 50.30 | 53.78 | 57.74 | 61.78 | 65.50 | 69.23 |
| 18.5 | 0.737 | 0.098 | 47.83 | 51.11 | 54.50 | 58.34 | 62.24 | 65.80 | 69.37 |
| 19.0 | 0.800 | 0.094 | 48.65 | 51.87 | 55.16 | 58.87 | 62.63 | 66.05 | 69.46 |
|  | Body cell mass (kg) | | | | | | | | |
| 10.0 | 0.004 | 0.194 | 9.04 | 10.16 | 11.42 | 13.02 | 14.84 | 16.69 | 18.74 |
| 10.5 | 0.046 | 0.192 | 9.53 | 10.71 | 12.04 | 13.71 | 15.60 | 17.51 | 19.62 |
| 11.0 | 0.095 | 0.190 | 10.15 | 11.41 | 12.83 | 14.59 | 16.57 | 18.56 | 20.73 |
| 11.5 | 0.129 | 0.188 | 10.76 | 12.10 | 13.60 | 15.45 | 17.52 | 19.59 | 21.83 |
| 12.0 | 0.156 | 0.186 | 11.51 | 12.93 | 14.53 | 16.49 | 18.67 | 20.84 | 23.18 |
| 12.5 | 0.177 | 0.184 | 12.39 | 13.92 | 15.62 | 17.71 | 20.02 | 22.31 | 24.77 |
| 13.0 | 0.198 | 0.181 | 13.40 | 15.04 | 16.86 | 19.08 | 21.54 | 23.95 | 26.55 |
| 13.5 | 0.232 | 0.178 | 14.71 | 16.48 | 18.44 | 20.83 | 23.44 | 26.00 | 28.74 |
| 14.0 | 0.272 | 0.174 | 15.81 | 17.70 | 19.77 | 22.27 | 24.99 | 27.65 | 30.46 |
| 14.5 | 0.320 | 0.169 | 16.91 | 18.90 | 21.06 | 23.66 | 26.47 | 29.19 | 32.05 |
| 15.0 | 0.370 | 0.164 | 18.01 | 20.08 | 22.33 | 25.00 | 27.87 | 30.62 | 33.49 |
| 15.5 | 0.429 | 0.157 | 19.31 | 21.46 | 23.77 | 26.50 | 29.40 | 32.16 | 35.02 |
| 16.0 | 0.471 | 0.152 | 20.35 | 22.55 | 24.89 | 27.64 | 30.54 | 33.29 | 36.12 |
| 16.5 | 0.506 | 0.146 | 21.33 | 23.55 | 25.91 | 28.66 | 31.54 | 34.25 | 37.04 |
| 17.0 | 0.535 | 0.140 | 22.22 | 24.44 | 26.79 | 29.52 | 32.37 | 35.04 | 37.78 |
| 17.5 | 0.562 | 0.135 | 23.01 | 25.23 | 27.56 | 30.26 | 33.06 | 35.68 | 38.35 |
| 18.0 | 0.596 | 0.129 | 23.87 | 26.07 | 28.38 | 31.03 | 33.78 | 36.33 | 38.93 |
| 18.5 | 0.628 | 0.125 | 24.55 | 26.74 | 29.02 | 31.64 | 34.34 | 36.84 | 39.38 |
| 19.0 | 0.660 | 0.121 | 25.21 | 27.38 | 29.64 | 32.23 | 34.88 | 37.33 | 39.81 |
|  | Total body water (L) | | | | | | | | |
| 10.0 | -1.300 | 0.092 | 21.14 | 22.14 | 23.27 | 24.70 | 26.34 | 28.05 | 30.02 |
| 10.5 | -1.103 | 0.095 | 21.69 | 22.78 | 24.00 | 25.52 | 27.27 | 29.08 | 31.12 |
| 11.0 | -0.909 | 0.098 | 22.26 | 23.44 | 24.76 | 26.40 | 28.26 | 30.16 | 32.29 |
| 11.5 | -0.722 | 0.101 | 22.95 | 24.24 | 25.67 | 27.43 | 29.42 | 31.43 | 33.66 |
| 12.0 | -0.517 | 0.105 | 24.02 | 25.45 | 27.03 | 28.98 | 31.14 | 33.30 | 35.67 |
| 12.5 | -0.366 | 0.108 | 25.08 | 26.65 | 28.39 | 30.49 | 32.82 | 35.13 | 37.63 |
| 13.0 | -0.231 | 0.110 | 26.26 | 27.98 | 29.86 | 32.14 | 34.64 | 37.10 | 39.73 |
| 13.5 | -0.109 | 0.112 | 27.45 | 29.32 | 31.35 | 33.80 | 36.46 | 39.05 | 41.81 |
| 14.0 | 0.008 | 0.113 | 28.57 | 30.57 | 32.74 | 35.33 | 38.13 | 40.84 | 43.69 |
| 14.5 | 0.124 | 0.113 | 29.55 | 31.68 | 33.97 | 36.69 | 39.59 | 42.38 | 45.29 |
| 15.0 | 0.242 | 0.113 | 30.42 | 32.66 | 35.05 | 37.86 | 40.84 | 43.67 | 46.61 |
| 15.5 | 0.360 | 0.113 | 31.16 | 33.49 | 35.96 | 38.84 | 41.87 | 44.71 | 47.64 |
| 16.0 | 0.471 | 0.111 | 31.81 | 34.21 | 36.74 | 39.66 | 42.70 | 45.54 | 48.44 |
| 16.5 | 0.566 | 0.110 | 32.40 | 34.86 | 37.42 | 40.36 | 43.40 | 46.22 | 49.07 |
| 17.0 | 0.656 | 0.107 | 33.09 | 35.57 | 38.15 | 41.09 | 44.10 | 46.88 | 49.67 |
| 17.5 | 0.723 | 0.105 | 33.62 | 36.11 | 38.68 | 41.60 | 44.58 | 47.31 | 50.04 |
| 18.0 | 0.792 | 0.103 | 34.10 | 36.60 | 39.16 | 42.05 | 44.99 | 47.66 | 50.33 |
| 18.5 | 0.865 | 0.100 | 34.55 | 37.04 | 39.60 | 42.46 | 45.34 | 47.96 | 50.57 |
| 19.0 | 0.942 | 0.098 | 34.98 | 37.47 | 40.01 | 42.84 | 45.68 | 48.24 | 50.78 |
|  | Muscle mass (kg) | | | | | | | | |
| 10.0 | -0.033 | 0.188 | 11.4 | 12.8 | 14.3 | 16.2 | 18.4 | 20.6 | 23.1 |
| 10.5 | 0.015 | 0.186 | 12.0 | 13.5 | 15.1 | 17.1 | 19.4 | 21.7 | 24.3 |
| 11.0 | 0.060 | 0.185 | 12.7 | 14.2 | 15.9 | 18.0 | 20.4 | 22.8 | 25.5 |
| 11.5 | 0.102 | 0.184 | 13.4 | 15.1 | 16.9 | 19.1 | 21.6 | 24.1 | 26.8 |
| 12.0 | 0.142 | 0.182 | 14.6 | 16.3 | 18.3 | 20.7 | 23.4 | 26.0 | 28.9 |
| 12.5 | 0.170 | 0.180 | 15.7 | 17.6 | 19.7 | 22.3 | 25.1 | 27.9 | 31.0 |
| 13.0 | 0.199 | 0.178 | 17.0 | 19.0 | 21.3 | 24.0 | 27.1 | 30.0 | 33.2 |
| 13.5 | 0.236 | 0.174 | 18.4 | 20.6 | 23.0 | 25.9 | 29.1 | 32.2 | 35.5 |
| 14.0 | 0.283 | 0.170 | 19.8 | 22.1 | 24.6 | 27.7 | 31.0 | 34.2 | 37.6 |
| 14.5 | 0.340 | 0.166 | 21.2 | 23.6 | 26.3 | 29.4 | 32.8 | 36.1 | 39.6 |
| 15.0 | 0.399 | 0.160 | 22.5 | 25.1 | 27.8 | 31.1 | 34.5 | 37.9 | 41.3 |
| 15.5 | 0.455 | 0.154 | 23.9 | 26.5 | 29.3 | 32.6 | 36.1 | 39.4 | 42.8 |
| 16.0 | 0.505 | 0.148 | 25.2 | 27.9 | 30.7 | 34.0 | 37.5 | 40.8 | 44.1 |
| 16.5 | 0.544 | 0.141 | 26.5 | 29.2 | 32.0 | 35.3 | 38.7 | 41.9 | 45.2 |
| 17.0 | 0.576 | 0.133 | 27.8 | 30.5 | 33.3 | 36.5 | 39.9 | 43.0 | 46.2 |
| 17.5 | 0.598 | 0.127 | 28.8 | 31.5 | 34.2 | 37.4 | 40.6 | 43.7 | 46.7 |
| 18.0 | 0.620 | 0.122 | 29.7 | 32.3 | 35.0 | 38.1 | 41.3 | 44.2 | 47.2 |
| 18.5 | 0.644 | 0.117 | 30.6 | 33.1 | 35.7 | 38.7 | 41.8 | 44.7 | 47.6 |
| 19.0 | 0.668 | 0.112 | 31.4 | 33.8 | 36.4 | 39.3 | 42.3 | 45.1 | 47.9 |
|  | Fat mass (kg) | | | | | | | | |
| 10.0 | 0.245 | 0.391 | 2.75 | 3.63 | 4.72 | 6.20 | 8.00 | 9.94 | 12.19 |
| 10.5 | 0.260 | 0.391 | 2.88 | 3.81 | 4.96 | 6.52 | 8.41 | 10.44 | 12.77 |
| 11.0 | 0.279 | 0.390 | 3.05 | 4.05 | 5.27 | 6.93 | 8.93 | 11.07 | 13.51 |
| 11.5 | 0.295 | 0.389 | 3.19 | 4.24 | 5.54 | 7.28 | 9.38 | 11.60 | 14.13 |
| 12.0 | 0.311 | 0.389 | 3.32 | 4.43 | 5.79 | 7.61 | 9.79 | 12.10 | 14.71 |
| 12.5 | 0.327 | 0.388 | 3.44 | 4.60 | 6.02 | 7.91 | 10.17 | 12.54 | 15.22 |
| 13.0 | 0.343 | 0.387 | 3.54 | 4.75 | 6.23 | 8.19 | 10.52 | 12.95 | 15.69 |
| 13.5 | 0.362 | 0.386 | 3.66 | 4.93 | 6.47 | 8.51 | 10.92 | 13.42 | 16.22 |
| 14.0 | 0.378 | 0.386 | 3.76 | 5.07 | 6.67 | 8.77 | 11.24 | 13.80 | 16.66 |
| 14.5 | 0.394 | 0.385 | 3.85 | 5.21 | 6.86 | 9.03 | 11.56 | 14.17 | 17.07 |
| 15.0 | 0.410 | 0.385 | 3.93 | 5.35 | 7.05 | 9.27 | 11.86 | 14.52 | 17.47 |
| 15.5 | 0.429 | 0.384 | 4.03 | 5.51 | 7.27 | 9.57 | 12.23 | 14.95 | 17.95 |
| 16.0 | 0.445 | 0.383 | 4.12 | 5.65 | 7.47 | 9.83 | 12.56 | 15.33 | 18.37 |
| 16.5 | 0.460 | 0.382 | 4.22 | 5.80 | 7.69 | 10.11 | 12.91 | 15.73 | 18.83 |
| 17.0 | 0.476 | 0.382 | 4.33 | 5.97 | 7.92 | 10.43 | 13.29 | 16.18 | 19.33 |
| 17.5 | 0.492 | 0.381 | 4.45 | 6.16 | 8.18 | 10.76 | 13.71 | 16.68 | 19.89 |
| 18.0 | 0.511 | 0.380 | 4.60 | 6.39 | 8.51 | 11.20 | 14.25 | 17.30 | 20.60 |
| 18.5 | 0.527 | 0.380 | 4.72 | 6.59 | 8.79 | 11.57 | 14.71 | 17.84 | 21.21 |
| 19.0 | 0.543 | 0.379 | 4.85 | 6.79 | 9.07 | 11.94 | 15.17 | 18.38 | 21.82 |
|  | Fat mass (%) | | | | | | | | |
| 10.0 | 0.571 | 0.323 | 8.97 | 11.79 | 14.97 | 18.89 | 23.19 | 27.38 | 31.81 |
| 10.5 | 0.581 | 0.324 | 8.81 | 11.61 | 14.78 | 18.66 | 22.92 | 27.06 | 31.42 |
| 11.0 | 0.593 | 0.325 | 8.61 | 11.40 | 14.54 | 18.38 | 22.59 | 26.67 | 30.96 |
| 11.5 | 0.603 | 0.326 | 8.45 | 11.22 | 14.34 | 18.15 | 22.31 | 26.34 | 30.57 |
| 12.0 | 0.613 | 0.327 | 8.28 | 11.03 | 14.12 | 17.90 | 22.01 | 25.99 | 30.16 |
| 12.5 | 0.623 | 0.328 | 8.10 | 10.83 | 13.90 | 17.63 | 21.69 | 25.61 | 29.72 |
| 13.0 | 0.633 | 0.329 | 7.92 | 10.63 | 13.67 | 17.36 | 21.36 | 25.22 | 29.26 |
| 13.5 | 0.644 | 0.330 | 7.71 | 10.39 | 13.40 | 17.04 | 20.98 | 24.76 | 28.72 |
| 14.0 | 0.654 | 0.331 | 7.54 | 10.21 | 13.18 | 16.78 | 20.67 | 24.40 | 28.30 |
| 14.5 | 0.664 | 0.332 | 7.38 | 10.03 | 12.98 | 16.55 | 20.39 | 24.07 | 27.91 |
| 15.0 | 0.674 | 0.333 | 7.23 | 9.87 | 12.80 | 16.34 | 20.14 | 23.78 | 27.56 |
| 15.5 | 0.686 | 0.335 | 7.08 | 9.71 | 12.63 | 16.13 | 19.90 | 23.49 | 27.22 |
| 16.0 | 0.696 | 0.336 | 6.97 | 9.60 | 12.51 | 16.01 | 19.75 | 23.32 | 27.01 |
| 16.5 | 0.706 | 0.337 | 6.88 | 9.52 | 12.43 | 15.92 | 19.66 | 23.20 | 26.87 |
| 17.0 | 0.716 | 0.338 | 6.81 | 9.46 | 12.39 | 15.89 | 19.62 | 23.16 | 26.81 |
| 17.5 | 0.726 | 0.339 | 6.75 | 9.43 | 12.37 | 15.89 | 19.63 | 23.17 | 26.82 |
| 18.0 | 0.738 | 0.340 | 6.70 | 9.41 | 12.39 | 15.92 | 19.68 | 23.24 | 26.89 |
| 18.5 | 0.748 | 0.341 | 6.67 | 9.41 | 12.41 | 15.97 | 19.75 | 23.31 | 26.97 |
| 19.0 | 0.758 | 0.342 | 6.63 | 9.40 | 12.43 | 16.02 | 19.82 | 23.39 | 27.06 |
|  | Fat-free mass index (kg/m^2^) | | | | | | | | |
| 10.0 | 0.674 | 0.079 | 11.61 | 12.22 | 12.86 | 13.58 | 14.31 | 14.97 | 15.64 |
| 10.5 | 0.659 | 0.080 | 11.80 | 12.43 | 13.08 | 13.82 | 14.57 | 15.26 | 15.94 |
| 11.0 | 0.643 | 0.080 | 11.98 | 12.62 | 13.29 | 14.05 | 14.82 | 15.52 | 16.23 |
| 11.5 | 0.627 | 0.081 | 12.20 | 12.86 | 13.55 | 14.32 | 15.12 | 15.85 | 16.58 |
| 12.0 | 0.615 | 0.082 | 12.40 | 13.08 | 13.79 | 14.59 | 15.40 | 16.15 | 16.91 |
| 12.5 | 0.604 | 0.083 | 12.64 | 13.34 | 14.06 | 14.88 | 15.72 | 16.50 | 17.27 |
| 13.0 | 0.594 | 0.084 | 12.89 | 13.61 | 14.35 | 15.20 | 16.07 | 16.86 | 17.66 |
| 13.5 | 0.581 | 0.084 | 13.15 | 13.89 | 14.65 | 15.52 | 16.41 | 17.23 | 18.06 |
| 14.0 | 0.560 | 0.085 | 13.40 | 14.16 | 14.94 | 15.83 | 16.75 | 17.59 | 18.44 |
| 14.5 | 0.519 | 0.085 | 13.70 | 14.47 | 15.28 | 16.19 | 17.13 | 18.00 | 18.88 |
| 15.0 | 0.471 | 0.085 | 13.96 | 14.74 | 15.56 | 16.49 | 17.45 | 18.34 | 19.24 |
| 15.5 | 0.408 | 0.085 | 14.22 | 15.01 | 15.84 | 16.78 | 17.76 | 18.67 | 19.60 |
| 16.0 | 0.331 | 0.085 | 14.48 | 15.27 | 16.10 | 17.06 | 18.06 | 18.99 | 19.93 |
| 16.5 | 0.243 | 0.085 | 14.72 | 15.51 | 16.35 | 17.32 | 18.32 | 19.27 | 20.24 |
| 17.0 | 0.154 | 0.084 | 14.93 | 15.72 | 16.56 | 17.53 | 18.55 | 19.51 | 20.50 |
| 17.5 | 0.075 | 0.084 | 15.09 | 15.88 | 16.72 | 17.70 | 18.73 | 19.71 | 20.71 |
| 18.0 | 0.003 | 0.084 | 15.24 | 16.03 | 16.87 | 17.85 | 18.89 | 19.88 | 20.91 |
| 18.5 | -0.041 | 0.084 | 15.33 | 16.12 | 16.96 | 17.95 | 18.99 | 19.99 | 21.03 |
| 19.0 | -0.077 | 0.084 | 15.41 | 16.20 | 17.04 | 18.03 | 19.08 | 20.09 | 21.14 |
|  | Body cell mass index (kg/m^2^) | | | | | | | | |
| 10.0 | 0.066 | 0.139 | 4.98 | 5.42 | 5.90 | 6.48 | 7.12 | 7.74 | 8.40 |
| 10.5 | 0.085 | 0.138 | 5.09 | 5.54 | 6.03 | 6.62 | 7.26 | 7.89 | 8.56 |
| 11.0 | 0.105 | 0.137 | 5.20 | 5.66 | 6.16 | 6.76 | 7.41 | 8.04 | 8.71 |
| 11.5 | 0.126 | 0.136 | 5.32 | 5.78 | 6.29 | 6.90 | 7.56 | 8.20 | 8.88 |
| 12.0 | 0.152 | 0.135 | 5.47 | 5.95 | 6.47 | 7.10 | 7.77 | 8.42 | 9.11 |
| 12.5 | 0.172 | 0.135 | 5.62 | 6.11 | 6.64 | 7.28 | 7.96 | 8.63 | 9.32 |
| 13.0 | 0.186 | 0.134 | 5.78 | 6.28 | 6.83 | 7.48 | 8.18 | 8.85 | 9.56 |
| 13.5 | 0.192 | 0.133 | 5.96 | 6.47 | 7.03 | 7.70 | 8.41 | 9.10 | 9.82 |
| 14.0 | 0.187 | 0.132 | 6.15 | 6.68 | 7.25 | 7.92 | 8.65 | 9.35 | 10.09 |
| 14.5 | 0.169 | 0.130 | 6.35 | 6.89 | 7.47 | 8.16 | 8.90 | 9.61 | 10.37 |
| 15.0 | 0.140 | 0.129 | 6.56 | 7.10 | 7.69 | 8.39 | 9.14 | 9.87 | 10.64 |
| 15.5 | 0.105 | 0.127 | 6.77 | 7.32 | 7.91 | 8.62 | 9.38 | 10.12 | 10.91 |
| 16.0 | 0.070 | 0.125 | 6.97 | 7.52 | 8.12 | 8.84 | 9.61 | 10.36 | 11.16 |
| 16.5 | 0.037 | 0.123 | 7.16 | 7.71 | 8.32 | 9.04 | 9.82 | 10.58 | 11.39 |
| 17.0 | 0.006 | 0.122 | 7.37 | 7.93 | 8.53 | 9.26 | 10.06 | 10.83 | 11.64 |
| 17.5 | -0.010 | 0.121 | 7.52 | 8.08 | 8.70 | 9.43 | 10.23 | 11.01 | 11.84 |
| 18.0 | -0.014 | 0.120 | 7.66 | 8.23 | 8.85 | 9.59 | 10.40 | 11.18 | 12.01 |
| 18.5 | -0.011 | 0.119 | 7.79 | 8.37 | 8.99 | 9.74 | 10.56 | 11.35 | 12.18 |
| 19.0 | -0.004 | 0.118 | 7.92 | 8.50 | 9.13 | 9.89 | 10.71 | 11.50 | 12.35 |
|  | Fat mass index (kg/m^2^) | | | | | | | | |
| 10.0 | 0.376 | 0.339 | 1.54 | 1.98 | 2.51 | 3.19 | 3.97 | 4.77 | 5.65 |
| 10.5 | 0.384 | 0.348 | 1.50 | 1.96 | 2.49 | 3.19 | 4.00 | 4.82 | 5.73 |
| 11.0 | 0.391 | 0.358 | 1.46 | 1.93 | 2.48 | 3.19 | 4.02 | 4.87 | 5.80 |
| 11.5 | 0.400 | 0.369 | 1.42 | 1.89 | 2.46 | 3.19 | 4.05 | 4.92 | 5.89 |
| 12.0 | 0.407 | 0.378 | 1.38 | 1.86 | 2.44 | 3.19 | 4.07 | 4.96 | 5.95 |
| 12.5 | 0.414 | 0.385 | 1.35 | 1.83 | 2.42 | 3.18 | 4.08 | 4.99 | 6.00 |
| 13.0 | 0.421 | 0.391 | 1.32 | 1.81 | 2.40 | 3.18 | 4.08 | 5.01 | 6.03 |
| 13.5 | 0.429 | 0.395 | 1.30 | 1.79 | 2.39 | 3.17 | 4.08 | 5.02 | 6.05 |
| 14.0 | 0.436 | 0.397 | 1.28 | 1.78 | 2.38 | 3.17 | 4.08 | 5.02 | 6.05 |
| 14.5 | 0.445 | 0.396 | 1.28 | 1.79 | 2.39 | 3.17 | 4.08 | 5.01 | 6.04 |
| 15.0 | 0.452 | 0.392 | 1.30 | 1.80 | 2.40 | 3.18 | 4.09 | 5.01 | 6.02 |
| 15.5 | 0.459 | 0.387 | 1.32 | 1.82 | 2.42 | 3.20 | 4.10 | 5.01 | 6.00 |
| 16.0 | 0.466 | 0.381 | 1.35 | 1.86 | 2.46 | 3.23 | 4.12 | 5.01 | 5.99 |
| 16.5 | 0.474 | 0.374 | 1.39 | 1.90 | 2.50 | 3.27 | 4.14 | 5.03 | 5.99 |
| 17.0 | 0.481 | 0.367 | 1.43 | 1.95 | 2.55 | 3.31 | 4.19 | 5.06 | 6.01 |
| 17.5 | 0.490 | 0.359 | 1.49 | 2.01 | 2.61 | 3.38 | 4.25 | 5.12 | 6.06 |
| 18.0 | 0.497 | 0.353 | 1.54 | 2.06 | 2.67 | 3.44 | 4.31 | 5.17 | 6.10 |
| 18.5 | 0.504 | 0.347 | 1.59 | 2.12 | 2.73 | 3.51 | 4.37 | 5.23 | 6.16 |
| 19.0 | 0.512 | 0.341 | 1.64 | 2.18 | 2.80 | 3.57 | 4.44 | 5.30 | 6.22 |

Abbreviations: L, lambda - Box-Cox transformation; M, median; S, sigma - coefficient of variation.
