# Supplementary material for: Reference data for body composition parameters in normal-weight Polish adolescents: results from the population-based ADOPOLNOR study
Source: Eur J Pediatr. 2024 Sep 26;183(11):5021–31. doi: 10.1007/s00431-024-05736-8 (PMC11473596; doi:10.1007/s00431-024-05736-8)
Supplement: Supplementary file 3 — Supplementary file3 (DOCX 92.2 KB) [file 431_2024_5736_MOESM3_ESM.docx]

Table S2. Centile reference values for body composition parameters in normal-weight Polish girls aged 10 to 18

| Age | LMS parameters | | Centiles | | | | | | |
| --- | --- | --- | --- | --- | --- | --- | --- | --- | --- |
| (years) | L | S | 3rd | 10th | 25th | M 50th | 75th | 90th | 97th |
|  | Fat-free mass (kg) | | | | | | | | |
| 10.0 | 0.541 | 0.154 | 20.00 | 22.24 | 24.62 | 27.39 | 30.30 | 33.03 | 35.83 |
| 10.5 | 0.490 | 0.150 | 21.34 | 23.63 | 26.06 | 28.90 | 31.89 | 34.72 | 37.62 |
| 11.0 | 0.429 | 0.145 | 22.97 | 25.30 | 27.78 | 30.70 | 33.78 | 36.70 | 39.72 |
| 11.5 | 0.377 | 0.141 | 24.34 | 26.69 | 29.21 | 32.17 | 35.31 | 38.30 | 41.40 |
| 12.0 | 0.326 | 0.136 | 25.71 | 28.07 | 30.61 | 33.60 | 36.78 | 39.82 | 42.97 |
| 12.5 | 0.275 | 0.131 | 27.07 | 29.42 | 31.96 | 34.96 | 38.16 | 41.22 | 44.41 |
| 13.0 | 0.224 | 0.126 | 28.37 | 30.71 | 33.24 | 36.23 | 39.42 | 42.48 | 45.68 |
| 13.5 | 0.162 | 0.121 | 29.83 | 32.14 | 34.64 | 37.59 | 40.76 | 43.79 | 46.97 |
| 14.0 | 0.111 | 0.116 | 30.94 | 33.21 | 35.67 | 38.58 | 41.70 | 44.70 | 47.85 |
| 14.5 | 0.060 | 0.111 | 31.93 | 34.16 | 36.57 | 39.43 | 42.49 | 45.44 | 48.55 |
| 15.0 | 0.008 | 0.107 | 32.81 | 34.99 | 37.34 | 40.14 | 43.15 | 46.05 | 49.10 |
| 15.5 | -0.053 | 0.103 | 33.71 | 35.82 | 38.11 | 40.84 | 43.77 | 46.60 | 49.58 |
| 16.0 | -0.104 | 0.099 | 34.33 | 36.40 | 38.63 | 41.30 | 44.16 | 46.93 | 49.86 |
| 16.5 | -0.156 | 0.096 | 34.85 | 36.87 | 39.05 | 41.65 | 44.46 | 47.18 | 50.04 |
| 17.0 | -0.207 | 0.094 | 35.26 | 37.24 | 39.37 | 41.93 | 44.68 | 47.34 | 50.17 |
| 17.5 | -0.258 | 0.092 | 35.60 | 37.53 | 39.63 | 42.13 | 44.84 | 47.46 | 50.25 |
| 18.0 | -0.320 | 0.089 | 35.92 | 37.81 | 39.87 | 42.32 | 44.98 | 47.56 | 50.31 |
| 18.5 | -0.371 | 0.088 | 36.16 | 38.02 | 40.04 | 42.45 | 45.07 | 47.63 | 50.34 |
| 19.0 | -0.422 | 0.086 | 36.39 | 38.22 | 40.20 | 42.58 | 45.17 | 47.69 | 50.38 |
|  | Body cell mass (kg) | | | | | | | | |
| 10.0 | -0.648 | 0.202 | 9.28 | 10.26 | 11.44 | 13.03 | 15.02 | 17.29 | 20.14 |
| 10.5 | -0.556 | 0.198 | 9.87 | 10.92 | 12.17 | 13.84 | 15.91 | 18.20 | 21.02 |
| 11.0 | -0.453 | 0.194 | 10.58 | 11.71 | 13.05 | 14.82 | 16.95 | 19.28 | 22.06 |
| 11.5 | -0.382 | 0.189 | 11.19 | 12.38 | 13.79 | 15.62 | 17.81 | 20.15 | 22.91 |
| 12.0 | -0.330 | 0.185 | 11.81 | 13.06 | 14.52 | 16.41 | 18.64 | 21.00 | 23.73 |
| 12.5 | -0.296 | 0.179 | 12.45 | 13.75 | 15.25 | 17.17 | 19.42 | 21.79 | 24.50 |
| 13.0 | -0.274 | 0.174 | 13.09 | 14.42 | 15.94 | 17.89 | 20.15 | 22.51 | 25.19 |
| 13.5 | -0.252 | 0.166 | 13.82 | 15.18 | 16.72 | 18.68 | 20.93 | 23.26 | 25.88 |
| 14.0 | -0.232 | 0.160 | 14.39 | 15.76 | 17.31 | 19.26 | 21.50 | 23.78 | 26.34 |
| 14.5 | -0.206 | 0.155 | 14.90 | 16.28 | 17.83 | 19.77 | 21.98 | 24.21 | 26.70 |
| 15.0 | -0.172 | 0.150 | 15.36 | 16.74 | 18.29 | 20.21 | 22.38 | 24.56 | 26.97 |
| 15.5 | -0.120 | 0.144 | 15.82 | 17.20 | 18.75 | 20.65 | 22.76 | 24.88 | 27.19 |
| 16.0 | -0.067 | 0.140 | 16.13 | 17.52 | 19.06 | 20.94 | 23.02 | 25.08 | 27.30 |
| 16.5 | -0.005 | 0.136 | 16.40 | 17.79 | 19.32 | 21.18 | 23.22 | 25.22 | 27.37 |
| 17.0 | 0.060 | 0.133 | 16.61 | 18.01 | 19.54 | 21.38 | 23.38 | 25.33 | 27.41 |
| 17.5 | 0.122 | 0.130 | 16.80 | 18.20 | 19.73 | 21.55 | 23.51 | 25.42 | 27.43 |
| 18.0 | 0.189 | 0.127 | 17.02 | 18.42 | 19.93 | 21.73 | 23.65 | 25.51 | 27.44 |
| 18.5 | 0.239 | 0.124 | 17.19 | 18.59 | 20.09 | 21.87 | 23.76 | 25.57 | 27.46 |
| 19.0 | 0.287 | 0.122 | 17.37 | 18.76 | 20.26 | 22.01 | 23.87 | 25.64 | 27.48 |
|  | Total body water (L) | | | | | | | | |
| 10.0 | 0.996 | 0.112 | 17.93 | 19.46 | 21.01 | 22.74 | 24.46 | 26.02 | 27.55 |
| 10.5 | 0.812 | 0.109 | 18.85 | 20.35 | 21.88 | 23.62 | 25.37 | 26.97 | 28.57 |
| 11.0 | 0.594 | 0.106 | 19.94 | 21.40 | 22.92 | 24.66 | 26.45 | 28.10 | 29.78 |
| 11.5 | 0.416 | 0.103 | 20.83 | 22.26 | 23.77 | 25.51 | 27.32 | 29.02 | 30.75 |
| 12.0 | 0.239 | 0.101 | 21.69 | 23.10 | 24.59 | 26.33 | 28.16 | 29.89 | 31.68 |
| 12.5 | 0.063 | 0.098 | 22.52 | 23.89 | 25.37 | 27.10 | 28.95 | 30.71 | 32.55 |
| 13.0 | -0.103 | 0.095 | 23.28 | 24.63 | 26.08 | 27.81 | 29.67 | 31.46 | 33.34 |
| 13.5 | -0.274 | 0.093 | 24.09 | 25.41 | 26.85 | 28.57 | 30.43 | 32.24 | 34.16 |
| 14.0 | -0.379 | 0.091 | 24.66 | 25.96 | 27.39 | 29.10 | 30.96 | 32.78 | 34.73 |
| 14.5 | -0.446 | 0.089 | 25.12 | 26.42 | 27.84 | 29.55 | 31.41 | 33.24 | 35.20 |
| 15.0 | -0.478 | 0.088 | 25.49 | 26.79 | 28.21 | 29.92 | 31.78 | 33.61 | 35.58 |
| 15.5 | -0.483 | 0.088 | 25.82 | 27.13 | 28.55 | 30.27 | 32.14 | 33.97 | 35.94 |
| 16.0 | -0.467 | 0.087 | 26.02 | 27.33 | 28.76 | 30.49 | 32.37 | 34.21 | 36.18 |
| 16.5 | -0.440 | 0.088 | 26.15 | 27.48 | 28.92 | 30.66 | 32.55 | 34.40 | 36.37 |
| 17.0 | -0.407 | 0.088 | 26.23 | 27.57 | 29.03 | 30.78 | 32.69 | 34.55 | 36.53 |
| 17.5 | -0.369 | 0.089 | 26.27 | 27.63 | 29.10 | 30.87 | 32.80 | 34.67 | 36.67 |
| 18.0 | -0.319 | 0.089 | 26.28 | 27.66 | 29.16 | 30.96 | 32.90 | 34.80 | 36.81 |
| 18.5 | -0.276 | 0.090 | 26.27 | 27.68 | 29.20 | 31.02 | 32.98 | 34.89 | 36.91 |
| 19.0 | -0.234 | 0.091 | 26.26 | 27.69 | 29.23 | 31.07 | 33.06 | 34.99 | 37.02 |
|  | Muscle mass (kg) | | | | | | | | |
| 10.0 | -0.440 | 0.199 | 11.49 | 12.76 | 14.26 | 16.24 | 18.65 | 21.29 | 24.45 |
| 10.5 | -0.415 | 0.195 | 12.25 | 13.59 | 15.17 | 17.24 | 19.74 | 22.44 | 25.66 |
| 11.0 | -0.386 | 0.190 | 13.19 | 14.60 | 16.26 | 18.43 | 21.02 | 23.80 | 27.08 |
| 11.5 | -0.362 | 0.186 | 13.98 | 15.46 | 17.18 | 19.42 | 22.07 | 24.90 | 28.19 |
| 12.0 | -0.337 | 0.181 | 14.78 | 16.31 | 18.09 | 20.38 | 23.08 | 25.93 | 29.23 |
| 12.5 | -0.312 | 0.175 | 15.58 | 17.16 | 18.98 | 21.31 | 24.04 | 26.89 | 30.16 |
| 13.0 | -0.288 | 0.169 | 16.38 | 17.99 | 19.84 | 22.19 | 24.92 | 27.75 | 30.96 |
| 13.5 | -0.259 | 0.161 | 17.29 | 18.94 | 20.80 | 23.16 | 25.86 | 28.64 | 31.76 |
| 14.0 | -0.234 | 0.155 | 18.01 | 19.66 | 21.53 | 23.87 | 26.54 | 29.25 | 32.28 |
| 14.5 | -0.209 | 0.149 | 18.66 | 20.32 | 22.18 | 24.50 | 27.11 | 29.76 | 32.69 |
| 15.0 | -0.185 | 0.143 | 19.25 | 20.90 | 22.75 | 25.03 | 27.59 | 30.17 | 32.99 |
| 15.5 | -0.155 | 0.137 | 19.87 | 21.51 | 23.33 | 25.57 | 28.06 | 30.54 | 33.24 |
| 16.0 | -0.131 | 0.132 | 20.32 | 21.94 | 23.74 | 25.93 | 28.36 | 30.77 | 33.37 |
| 16.5 | -0.106 | 0.128 | 20.70 | 22.30 | 24.08 | 26.23 | 28.60 | 30.94 | 33.45 |
| 17.0 | -0.082 | 0.124 | 21.02 | 22.61 | 24.36 | 26.47 | 28.79 | 31.06 | 33.49 |
| 17.5 | -0.057 | 0.120 | 21.30 | 22.88 | 24.60 | 26.68 | 28.94 | 31.15 | 33.51 |
| 18.0 | -0.028 | 0.117 | 21.60 | 23.16 | 24.86 | 26.89 | 29.09 | 31.24 | 33.51 |
| 18.5 | -0.003 | 0.114 | 21.84 | 23.38 | 25.06 | 27.05 | 29.21 | 31.30 | 33.51 |
| 19.0 | 0.021 | 0.111 | 22.08 | 23.60 | 25.25 | 27.22 | 29.33 | 31.37 | 33.52 |
|  | Fat mass (kg) | | | | | | | | |
| 10.0 | 0.144 | 0.354 | 3.33 | 4.20 | 5.26 | 6.71 | 8.49 | 10.42 | 12.69 |
| 10.5 | 0.230 | 0.350 | 3.54 | 4.50 | 5.67 | 7.23 | 9.10 | 11.07 | 13.34 |
| 11.0 | 0.332 | 0.345 | 3.81 | 4.90 | 6.20 | 7.90 | 9.88 | 11.93 | 14.21 |
| 11.5 | 0.412 | 0.340 | 4.06 | 5.27 | 6.70 | 8.53 | 10.62 | 12.74 | 15.05 |
| 12.0 | 0.490 | 0.336 | 4.33 | 5.69 | 7.25 | 9.22 | 11.42 | 13.62 | 15.98 |
| 12.5 | 0.564 | 0.331 | 4.62 | 6.12 | 7.83 | 9.94 | 12.26 | 14.53 | 16.94 |
| 13.0 | 0.639 | 0.325 | 4.91 | 6.57 | 8.42 | 10.66 | 13.09 | 15.42 | 17.86 |
| 13.5 | 0.730 | 0.317 | 5.25 | 7.09 | 9.10 | 11.48 | 14.00 | 16.38 | 18.83 |
| 14.0 | 0.802 | 0.309 | 5.52 | 7.51 | 9.63 | 12.10 | 14.68 | 17.07 | 19.51 |
| 14.5 | 0.864 | 0.302 | 5.82 | 7.92 | 10.13 | 12.67 | 15.28 | 17.69 | 20.10 |
| 15.0 | 0.910 | 0.294 | 6.13 | 8.33 | 10.61 | 13.20 | 15.84 | 18.25 | 20.65 |
| 15.5 | 0.944 | 0.285 | 6.54 | 8.81 | 11.15 | 13.78 | 16.44 | 18.86 | 21.26 |
| 16.0 | 0.952 | 0.278 | 6.89 | 9.19 | 11.56 | 14.22 | 16.90 | 19.33 | 21.74 |
| 16.5 | 0.945 | 0.273 | 7.24 | 9.55 | 11.93 | 14.61 | 17.31 | 19.76 | 22.20 |
| 17.0 | 0.927 | 0.268 | 7.58 | 9.89 | 12.27 | 14.96 | 17.68 | 20.16 | 22.63 |
| 17.5 | 0.901 | 0.264 | 7.90 | 10.20 | 12.59 | 15.29 | 18.04 | 20.55 | 23.06 |
| 18.0 | 0.867 | 0.261 | 8.28 | 10.57 | 12.95 | 15.67 | 18.46 | 21.02 | 23.58 |
| 18.5 | 0.837 | 0.258 | 8.58 | 10.86 | 13.25 | 15.99 | 18.81 | 21.41 | 24.02 |
| 19.0 | 0.808 | 0.255 | 8.88 | 11.15 | 13.55 | 16.31 | 19.16 | 21.80 | 24.46 |
|  | Fat mass (%) | | | | | | | | |
| 10.0 | 0.542 | 0.295 | 10.50 | 13.30 | 16.45 | 20.30 | 24.52 | 28.63 | 32.97 |
| 10.5 | 0.630 | 0.287 | 10.63 | 13.53 | 16.72 | 20.55 | 24.66 | 28.60 | 32.69 |
| 11.0 | 0.736 | 0.278 | 10.82 | 13.84 | 17.09 | 20.90 | 24.91 | 28.67 | 32.51 |
| 11.5 | 0.825 | 0.270 | 11.01 | 14.15 | 17.46 | 21.26 | 25.20 | 28.83 | 32.50 |
| 12.0 | 0.913 | 0.263 | 11.24 | 14.50 | 17.87 | 21.68 | 25.55 | 29.08 | 32.60 |
| 12.5 | 1.002 | 0.256 | 11.50 | 14.89 | 18.33 | 22.15 | 25.96 | 29.40 | 32.79 |
| 13.0 | 1.090 | 0.249 | 11.78 | 15.31 | 18.81 | 22.64 | 26.40 | 29.76 | 33.03 |
| 13.5 | 1.197 | 0.241 | 12.13 | 15.81 | 19.39 | 23.22 | 26.93 | 30.19 | 33.34 |
| 14.0 | 1.285 | 0.234 | 12.42 | 16.24 | 19.87 | 23.70 | 27.36 | 30.54 | 33.59 |
| 14.5 | 1.373 | 0.228 | 12.73 | 16.66 | 20.34 | 24.16 | 27.77 | 30.87 | 33.83 |
| 15.0 | 1.462 | 0.222 | 13.04 | 17.08 | 20.80 | 24.60 | 28.16 | 31.19 | 34.06 |
| 15.5 | 1.568 | 0.214 | 13.42 | 17.57 | 21.33 | 25.11 | 28.60 | 31.55 | 34.31 |
| 16.0 | 1.657 | 0.209 | 13.75 | 17.98 | 21.75 | 25.51 | 28.94 | 31.82 | 34.50 |
| 16.5 | 1.745 | 0.203 | 14.09 | 18.39 | 22.18 | 25.91 | 29.28 | 32.09 | 34.69 |
| 17.0 | 1.834 | 0.197 | 14.45 | 18.83 | 22.62 | 26.33 | 29.65 | 32.39 | 34.92 |
| 17.5 | 1.922 | 0.192 | 14.86 | 19.29 | 23.10 | 26.78 | 30.05 | 32.73 | 35.20 |
| 18.0 | 2.028 | 0.186 | 15.38 | 19.88 | 23.71 | 27.36 | 30.57 | 33.20 | 35.60 |
| 18.5 | 2.117 | 0.181 | 15.84 | 20.40 | 24.23 | 27.85 | 31.03 | 33.61 | 35.95 |
| 19.0 | 2.205 | 0.176 | 16.31 | 20.91 | 24.75 | 28.35 | 31.48 | 34.01 | 36.31 |
|  | Fat-free mass index (kg/m^2^) | | | | | | | | |
| 10.0 | -0.584 | 0.093 | 11.27 | 11.86 | 12.52 | 13.31 | 14.19 | 15.06 | 16.00 |
| 10.5 | -0.530 | 0.091 | 11.48 | 12.07 | 12.73 | 13.52 | 14.39 | 15.25 | 16.18 |
| 11.0 | -0.465 | 0.089 | 11.73 | 12.33 | 12.98 | 13.77 | 14.64 | 15.48 | 16.39 |
| 11.5 | -0.410 | 0.087 | 11.93 | 12.53 | 13.19 | 13.98 | 14.83 | 15.67 | 16.56 |
| 12.0 | -0.356 | 0.085 | 12.13 | 12.73 | 13.39 | 14.17 | 15.02 | 15.85 | 16.72 |
| 12.5 | -0.302 | 0.084 | 12.32 | 12.92 | 13.58 | 14.36 | 15.20 | 16.01 | 16.87 |
| 13.0 | -0.247 | 0.082 | 12.49 | 13.10 | 13.76 | 14.53 | 15.36 | 16.16 | 17.00 |
| 13.5 | -0.182 | 0.080 | 12.69 | 13.30 | 13.95 | 14.71 | 15.53 | 16.32 | 17.14 |
| 14.0 | -0.128 | 0.078 | 12.83 | 13.44 | 14.09 | 14.85 | 15.66 | 16.43 | 17.23 |
| 14.5 | -0.074 | 0.077 | 12.97 | 13.57 | 14.22 | 14.97 | 15.77 | 16.52 | 17.31 |
| 15.0 | -0.019 | 0.075 | 13.09 | 13.69 | 14.33 | 15.07 | 15.86 | 16.60 | 17.37 |
| 15.5 | 0.046 | 0.073 | 13.22 | 13.82 | 14.45 | 15.18 | 15.95 | 16.67 | 17.42 |
| 16.0 | 0.100 | 0.072 | 13.32 | 13.91 | 14.53 | 15.26 | 16.01 | 16.72 | 17.45 |
| 16.5 | 0.155 | 0.070 | 13.40 | 13.99 | 14.61 | 15.32 | 16.06 | 16.76 | 17.47 |
| 17.0 | 0.209 | 0.069 | 13.48 | 14.06 | 14.67 | 15.37 | 16.10 | 16.78 | 17.48 |
| 17.5 | 0.263 | 0.068 | 13.55 | 14.12 | 14.73 | 15.42 | 16.13 | 16.80 | 17.47 |
| 18.0 | 0.329 | 0.066 | 13.62 | 14.19 | 14.79 | 15.46 | 16.16 | 16.81 | 17.46 |
| 18.5 | 0.383 | 0.065 | 13.68 | 14.25 | 14.83 | 15.50 | 16.18 | 16.82 | 17.46 |
| 19.0 | 0.437 | 0.063 | 13.74 | 14.30 | 14.88 | 15.53 | 16.20 | 16.82 | 17.45 |
|  | Body cell mass index (kg/m^2^) | | | | | | | | |
| 10.0 | -1.193 | 0.158 | 4.96 | 5.34 | 5.79 | 6.40 | 7.17 | 8.06 | 9.23 |
| 10.5 | -1.101 | 0.155 | 5.07 | 5.45 | 5.91 | 6.53 | 7.29 | 8.17 | 9.28 |
| 11.0 | -0.989 | 0.152 | 5.19 | 5.59 | 6.06 | 6.68 | 7.44 | 8.30 | 9.35 |
| 11.5 | -0.897 | 0.150 | 5.30 | 5.71 | 6.18 | 6.81 | 7.57 | 8.40 | 9.42 |
| 12.0 | -0.804 | 0.147 | 5.40 | 5.82 | 6.30 | 6.94 | 7.69 | 8.51 | 9.48 |
| 12.5 | -0.711 | 0.145 | 5.51 | 5.93 | 6.42 | 7.06 | 7.81 | 8.61 | 9.55 |
| 13.0 | -0.619 | 0.142 | 5.60 | 6.04 | 6.54 | 7.18 | 7.92 | 8.71 | 9.62 |
| 13.5 | -0.508 | 0.139 | 5.72 | 6.16 | 6.67 | 7.31 | 8.05 | 8.82 | 9.69 |
| 14.0 | -0.415 | 0.137 | 5.80 | 6.26 | 6.77 | 7.41 | 8.15 | 8.90 | 9.74 |
| 14.5 | -0.322 | 0.135 | 5.88 | 6.35 | 6.86 | 7.51 | 8.23 | 8.96 | 9.78 |
| 15.0 | -0.230 | 0.132 | 5.96 | 6.42 | 6.95 | 7.59 | 8.30 | 9.02 | 9.81 |
| 15.5 | -0.118 | 0.130 | 6.03 | 6.51 | 7.03 | 7.67 | 8.38 | 9.08 | 9.83 |
| 16.0 | -0.026 | 0.128 | 6.09 | 6.57 | 7.10 | 7.73 | 8.43 | 9.11 | 9.84 |
| 16.5 | 0.067 | 0.125 | 6.14 | 6.62 | 7.15 | 7.79 | 8.47 | 9.14 | 9.84 |
| 17.0 | 0.160 | 0.123 | 6.18 | 6.68 | 7.20 | 7.83 | 8.51 | 9.16 | 9.84 |
| 17.5 | 0.252 | 0.121 | 6.23 | 6.72 | 7.25 | 7.88 | 8.54 | 9.18 | 9.83 |
| 18.0 | 0.363 | 0.119 | 6.28 | 6.78 | 7.31 | 7.93 | 8.58 | 9.19 | 9.83 |
| 18.5 | 0.456 | 0.117 | 6.32 | 6.82 | 7.35 | 7.97 | 8.61 | 9.21 | 9.83 |
| 19.0 | 0.549 | 0.115 | 6.36 | 6.87 | 7.40 | 8.01 | 8.64 | 9.23 | 9.82 |
|  | Fat mass index (kg/m^2^) | | | | | | | | |
| 10.0 | 0.125 | 0.331 | 1.76 | 2.17 | 2.68 | 3.36 | 4.18 | 5.08 | 6.11 |
| 10.5 | 0.243 | 0.327 | 1.78 | 2.23 | 2.77 | 3.47 | 4.30 | 5.17 | 6.16 |
| 11.0 | 0.380 | 0.323 | 1.82 | 2.31 | 2.89 | 3.62 | 4.47 | 5.32 | 6.25 |
| 11.5 | 0.488 | 0.319 | 1.85 | 2.39 | 3.00 | 3.77 | 4.62 | 5.47 | 6.38 |
| 12.0 | 0.589 | 0.315 | 1.89 | 2.48 | 3.13 | 3.93 | 4.80 | 5.64 | 6.53 |
| 12.5 | 0.685 | 0.311 | 1.94 | 2.57 | 3.27 | 4.10 | 4.98 | 5.82 | 6.69 |
| 13.0 | 0.776 | 0.306 | 1.99 | 2.68 | 3.41 | 4.27 | 5.17 | 6.01 | 6.87 |
| 13.5 | 0.879 | 0.298 | 2.06 | 2.81 | 3.58 | 4.47 | 5.38 | 6.22 | 7.05 |
| 14.0 | 0.954 | 0.292 | 2.13 | 2.92 | 3.72 | 4.63 | 5.55 | 6.37 | 7.20 |
| 14.5 | 1.014 | 0.284 | 2.22 | 3.04 | 3.87 | 4.79 | 5.70 | 6.53 | 7.34 |
| 15.0 | 1.054 | 0.277 | 2.32 | 3.17 | 4.01 | 4.93 | 5.85 | 6.67 | 7.48 |
| 15.5 | 1.074 | 0.269 | 2.47 | 3.32 | 4.17 | 5.10 | 6.02 | 6.84 | 7.64 |
| 16.0 | 1.067 | 0.262 | 2.60 | 3.45 | 4.30 | 5.24 | 6.16 | 6.98 | 7.78 |
| 16.5 | 1.041 | 0.257 | 2.74 | 3.58 | 4.43 | 5.36 | 6.28 | 7.11 | 7.92 |
| 17.0 | 0.999 | 0.252 | 2.88 | 3.70 | 4.54 | 5.47 | 6.40 | 7.24 | 8.07 |
| 17.5 | 0.947 | 0.248 | 3.01 | 3.82 | 4.65 | 5.58 | 6.52 | 7.37 | 8.22 |
| 18.0 | 0.879 | 0.244 | 3.17 | 3.96 | 4.79 | 5.72 | 6.67 | 7.54 | 8.41 |
| 18.5 | 0.819 | 0.241 | 3.30 | 4.08 | 4.90 | 5.83 | 6.79 | 7.68 | 8.58 |
| 19.0 | 0.760 | 0.239 | 3.43 | 4.20 | 5.01 | 5.94 | 6.92 | 7.82 | 8.75 |

Abbreviations: L, Lambda - Box-Cox transformation; M, median; S, sigma - coefficient of variation.
