# Supplementary figures and images for: Reference data for body composition parameters in normal-weight Polish adolescents: results from the population-based ADOPOLNOR study
Source: Eur J Pediatr. 2024 Sep 26;183(11):5021–31. doi: 10.1007/s00431-024-05736-8 (PMC11473596; doi:10.1007/s00431-024-05736-8)

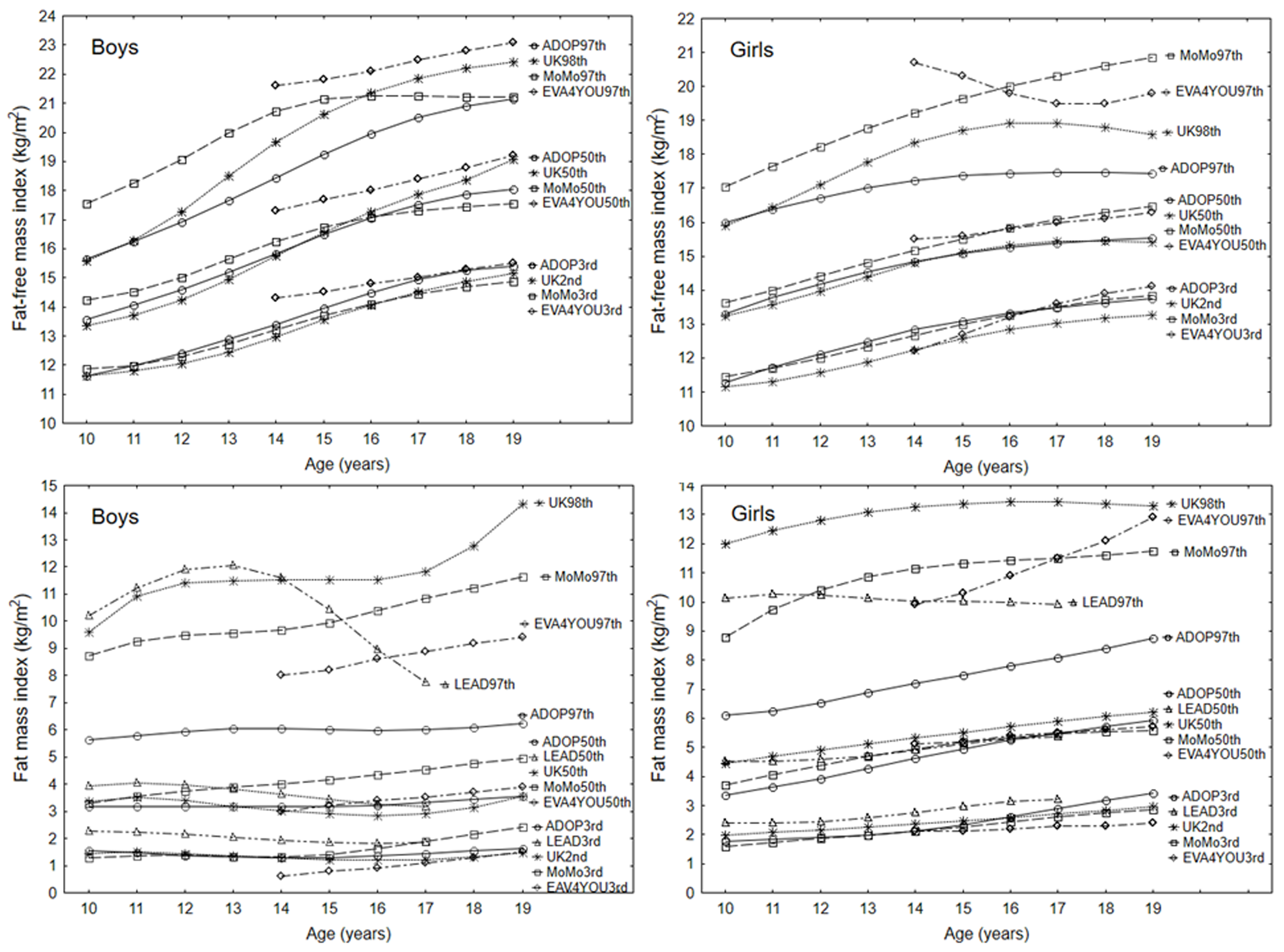

Supplement: Supplementary file 4 — Comparison of the raw 3rd (2nd in Britain cohort), 50th, and 97th (98th in Britain cohort) reference centile curves for FFMI and FMI in normal-weight Polish adolescents with population data from Austrian (LEAD) [15], United Kingdom [28], German (MoMoP) [16] and Tyrolean (EVA4YOU) adolescents [29]. (PNG 1740 KB) [file 431_2024_5736_Fig4_ESM.png]

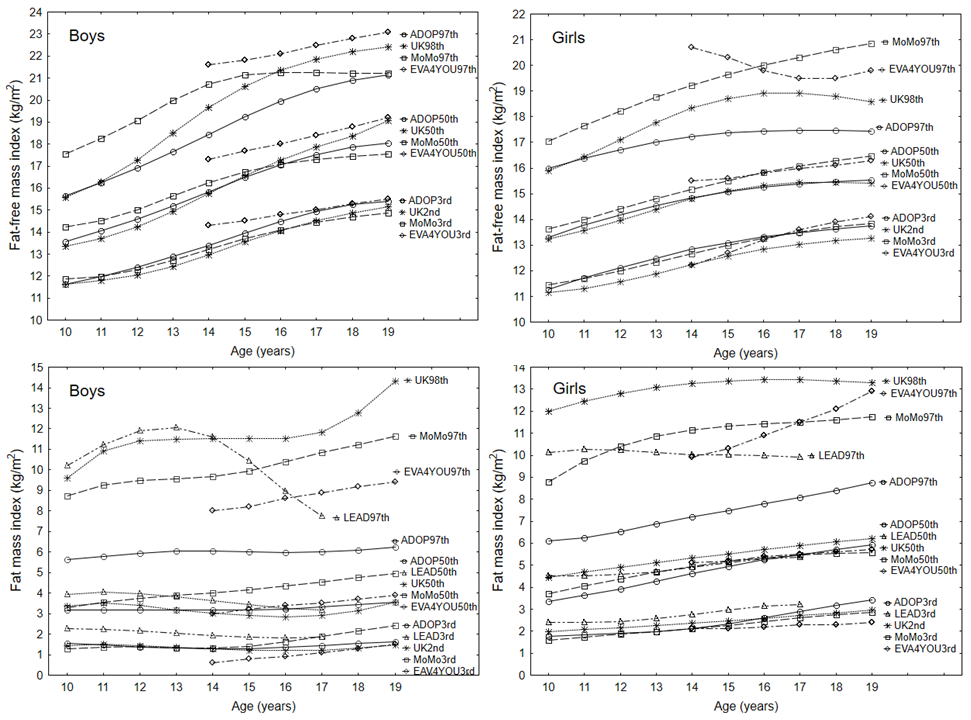

Supplement: Supplementary file 5 — High resolution image (TIF 306 kb) [file 431_2024_5736_MOESM4_ESM.tif]

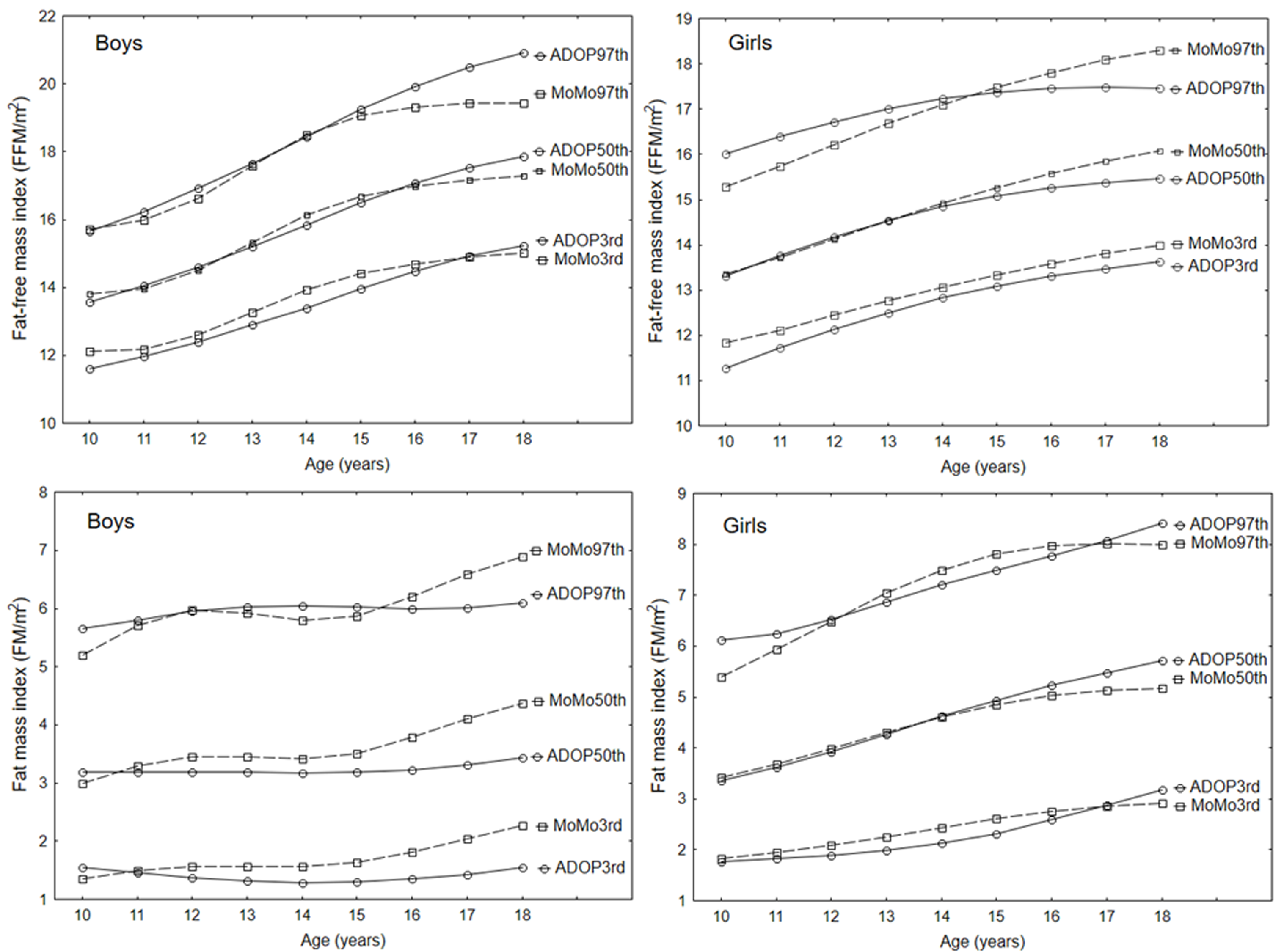

Supplement: Supplementary file 6 — Comparison of the raw 3rd, 50th, and 97th reference centile curves for FFMI and FMI in normal-weight Polish and normal-weight German adolescents (MoMoNW) [16]. (PNG 1130 KB) [file 431_2024_5736_Fig5_ESM.png]

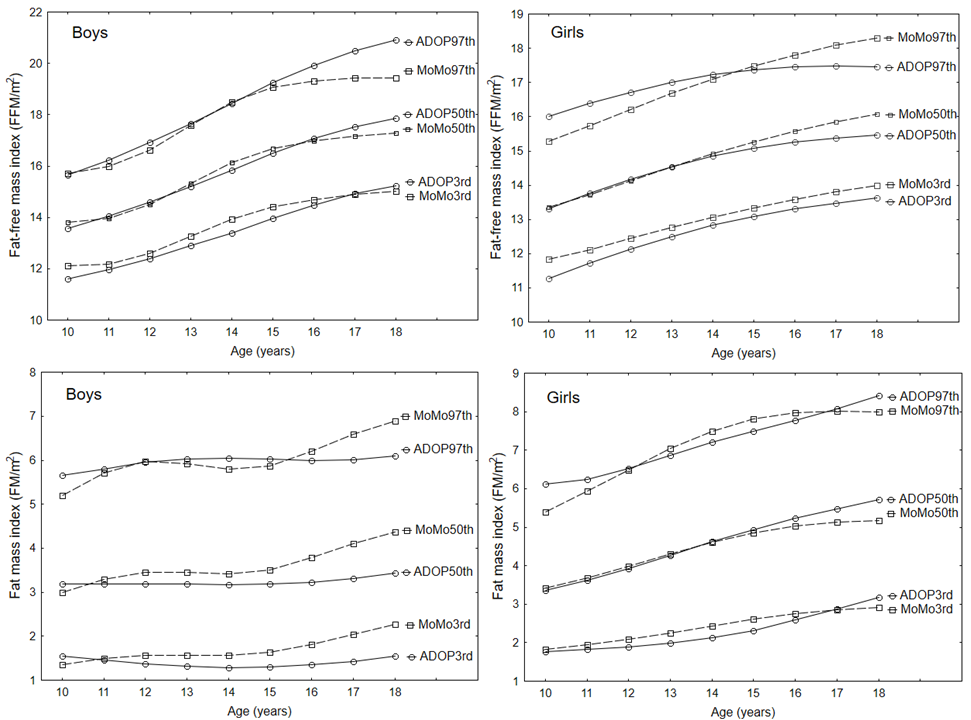

Supplement: Supplementary file 7 — High resolution image (TIF 211 kb) [file 431_2024_5736_MOESM5_ESM.tif]
